# Study of the $B_c^+ \rightarrow J/\psi D_s^+$ and $B_c^+ \rightarrow J/\psi D_s^{*+}$ decays with the ATLAS detector

**DOI:** 10.1140/epjc/s10052-015-3743-8

**Published:** 2016-01-05

**Authors:** G. Aad, B. Abbott, J. Abdallah, O. Abdinov, R. Aben, M. Abolins, O. S. AbouZeid, H. Abramowicz, H. Abreu, R. Abreu, Y. Abulaiti, B. S. Acharya, L. Adamczyk, D. L. Adams, J. Adelman, S. Adomeit, T. Adye, A. A. Affolder, T. Agatonovic-Jovin, J. A. Aguilar-Saavedra, S. P. Ahlen, F. Ahmadov, G. Aielli, H. Akerstedt, T. P. A. Åkesson, G. Akimoto, A. V. Akimov, G. L. Alberghi, J. Albert, S. Albrand, M. J. Alconada Verzini, M. Aleksa, I. N. Aleksandrov, C. Alexa, G. Alexander, T. Alexopoulos, M. Alhroob, G. Alimonti, L. Alio, J. Alison, S. P. Alkire, B. M. M. Allbrooke, P. P. Allport, A. Aloisio, A. Alonso, F. Alonso, C. Alpigiani, A. Altheimer, B. Alvarez Gonzalez, D. Álvarez Piqueras, M. G. Alviggi, B. T. Amadio, K. Amako, Y. Amaral Coutinho, C. Amelung, D. Amidei, S. P. Amor Dos Santos, A. Amorim, S. Amoroso, N. Amram, G. Amundsen, C. Anastopoulos, L. S. Ancu, N. Andari, T. Andeen, C. F. Anders, G. Anders, J. K. Anders, K. J. Anderson, A. Andreazza, V. Andrei, S. Angelidakis, I. Angelozzi, P. Anger, A. Angerami, F. Anghinolfi, A. V. Anisenkov, N. Anjos, A. Annovi, M. Antonelli, A. Antonov, J. Antos, F. Anulli, M. Aoki, L. Aperio Bella, G. Arabidze, Y. Arai, J. P. Araque, A. T. H. Arce, F. A. Arduh, J-F. Arguin, S. Argyropoulos, M. Arik, A. J. Armbruster, O. Arnaez, V. Arnal, H. Arnold, M. Arratia, O. Arslan, A. Artamonov, G. Artoni, S. Asai, N. Asbah, A. Ashkenazi, B. Åsman, L. Asquith, K. Assamagan, R. Astalos, M. Atkinson, N. B. Atlay, B. Auerbach, K. Augsten, M. Aurousseau, G. Avolio, B. Axen, M. K. Ayoub, G. Azuelos, M. A. Baak, A. E. Baas, C. Bacci, H. Bachacou, K. Bachas, M. Backes, M. Backhaus, P. Bagiacchi, P. Bagnaia, Y. Bai, T. Bain, J. T. Baines, O. K. Baker, P. Balek, T. Balestri, F. Balli, E. Banas, Sw. Banerjee, A. A. E. Bannoura, H. S. Bansil, L. Barak, E. L. Barberio, D. Barberis, M. Barbero, T. Barillari, M. Barisonzi, T. Barklow, N. Barlow, S. L. Barnes, B. M. Barnett, R. M. Barnett, Z. Barnovska, A. Baroncelli, G. Barone, A. J. Barr, F. Barreiro, J. Barreiro Guimarães da Costa, R. Bartoldus, A. E. Barton, P. Bartos, A. Basalaev, A. Bassalat, A. Basye, R. L. Bates, S. J. Batista, J. R. Batley, M. Battaglia, M. Bauce, F. Bauer, H. S. Bawa, J. B. Beacham, M. D. Beattie, T. Beau, P. H. Beauchemin, R. Beccherle, P. Bechtle, H. P. Beck, K. Becker, M. Becker, S. Becker, M. Beckingham, C. Becot, A. J. Beddall, A. Beddall, V. A. Bednyakov, C. P. Bee, L. J. Beemster, T. A. Beermann, M. Begel, J. K. Behr, C. Belanger-Champagne, W. H. Bell, G. Bella, L. Bellagamba, A. Bellerive, M. Bellomo, K. Belotskiy, O. Beltramello, O. Benary, D. Benchekroun, M. Bender, K. Bendtz, N. Benekos, Y. Benhammou, E. Benhar Noccioli, J. A. Benitez Garcia, D. P. Benjamin, J. R. Bensinger, S. Bentvelsen, L. Beresford, M. Beretta, D. Berge, E. Bergeaas Kuutmann, N. Berger, F. Berghaus, J. Beringer, C. Bernard, N. R. Bernard, C. Bernius, F. U. Bernlochner, T. Berry, P. Berta, C. Bertella, G. Bertoli, F. Bertolucci, C. Bertsche, D. Bertsche, M. I. Besana, G. J. Besjes, O. Bessidskaia Bylund, M. Bessner, N. Besson, C. Betancourt, S. Bethke, A. J. Bevan, W. Bhimji, R. M. Bianchi, L. Bianchini, M. Bianco, O. Biebel, S. P. Bieniek, M. Biglietti, J. Bilbao De Mendizabal, H. Bilokon, M. Bindi, S. Binet, A. Bingul, C. Bini, C. W. Black, J. E. Black, K. M. Black, D. Blackburn, R. E. Blair, J.-B. Blanchard, J. E. Blanco, T. Blazek, I. Bloch, C. Blocker, W. Blum, U. Blumenschein, G. J. Bobbink, V. S. Bobrovnikov, S. S. Bocchetta, A. Bocci, C. Bock, M. Boehler, J. A. Bogaerts, D. Bogavac, A. G. Bogdanchikov, C. Bohm, V. Boisvert, T. Bold, V. Boldea, A. S. Boldyrev, M. Bomben, M. Bona, M. Boonekamp, A. Borisov, G. Borissov, S. Borroni, J. Bortfeldt, V. Bortolotto, K. Bos, D. Boscherini, M. Bosman, J. Boudreau, J. Bouffard, E. V. Bouhova-Thacker, D. Boumediene, C. Bourdarios, N. Bousson, A. Boveia, J. Boyd, I. R. Boyko, I. Bozic, J. Bracinik, A. Brandt, G. Brandt, O. Brandt, U. Bratzler, B. Brau, J. E. Brau, H. M. Braun, S. F. Brazzale, W. D. Breaden Madden, K. Brendlinger, A. J. Brennan, L. Brenner, R. Brenner, S. Bressler, K. Bristow, T. M. Bristow, D. Britton, D. Britzger, F. M. Brochu, I. Brock, R. Brock, J. Bronner, G. Brooijmans, T. Brooks, W. K. Brooks, J. Brosamer, E. Brost, J. Brown, P. A. Bruckman de Renstrom, D. Bruncko, R. Bruneliere, A. Bruni, G. Bruni, M. Bruschi, N. Bruscino, L. Bryngemark, T. Buanes, Q. Buat, P. Buchholz, A. G. Buckley, S. I. Buda, I. A. Budagov, F. Buehrer, L. Bugge, M. K. Bugge, O. Bulekov, D. Bullock, H. Burckhart, S. Burdin, B. Burghgrave, S. Burke, I. Burmeister, E. Busato, D. Büscher, V. Büscher, P. Bussey, J. M. Butler, A. I. Butt, C. M. Buttar, J. M. Butterworth, P. Butti, W. Buttinger, A. Buzatu, A. R. Buzykaev, S. Cabrera Urbán, D. Caforio, V. M. Cairo, O. Cakir, P. Calafiura, A. Calandri, G. Calderini, P. Calfayan, L. P. Caloba, D. Calvet, S. Calvet, R. Camacho Toro, S. Camarda, P. Camarri, D. Cameron, L. M. Caminada, R. Caminal Armadans, S. Campana, M. Campanelli, A. Campoverde, V. Canale, A. Canepa, M. Cano Bret, J. Cantero, R. Cantrill, T. Cao, M. D. M. Capeans Garrido, I. Caprini, M. Caprini, M. Capua, R. Caputo, R. Cardarelli, F. Cardillo, T. Carli, G. Carlino, L. Carminati, S. Caron, E. Carquin, G. D. Carrillo-Montoya, J. R. Carter, J. Carvalho, D. Casadei, M. P. Casado, M. Casolino, E. Castaneda-Miranda, A. Castelli, V. Castillo Gimenez, N. F. Castro, P. Catastini, A. Catinaccio, J. R. Catmore, A. Cattai, J. Caudron, V. Cavaliere, D. Cavalli, M. Cavalli-Sforza, V. Cavasinni, F. Ceradini, B. C. Cerio, K. Cerny, A. S. Cerqueira, A. Cerri, L. Cerrito, F. Cerutti, M. Cerv, A. Cervelli, S. A. Cetin, A. Chafaq, D. Chakraborty, I. Chalupkova, P. Chang, B. Chapleau, J. D. Chapman, D. G. Charlton, C. C. Chau, C. A. Chavez Barajas, S. Cheatham, A. Chegwidden, S. Chekanov, S. V. Chekulaev, G. A. Chelkov, M. A. Chelstowska, C. Chen, H. Chen, K. Chen, L. Chen, S. Chen, X. Chen, Y. Chen, H. C. Cheng, Y. Cheng, A. Cheplakov, E. Cheremushkina, R. Cherkaoui El Moursli, V. Chernyatin, E. Cheu, L. Chevalier, V. Chiarella, J. T. Childers, G. Chiodini, A. S. Chisholm, R. T. Chislett, A. Chitan, M. V. Chizhov, K. Choi, S. Chouridou, B. K. B. Chow, V. Christodoulou, D. Chromek-Burckhart, J. Chudoba, A. J. Chuinard, J. J. Chwastowski, L. Chytka, G. Ciapetti, A. K. Ciftci, D. Cinca, V. Cindro, I. A. Cioara, A. Ciocio, Z. H. Citron, M. Ciubancan, A. Clark, B. L. Clark, P. J. Clark, R. N. Clarke, W. Cleland, C. Clement, Y. Coadou, M. Cobal, A. Coccaro, J. Cochran, L. Coffey, J. G. Cogan, B. Cole, S. Cole, A. P. Colijn, J. Collot, T. Colombo, G. Compostella, P. Conde Muiño, E. Coniavitis, S. H. Connell, I. A. Connelly, S. M. Consonni, V. Consorti, S. Constantinescu, C. Conta, G. Conti, F. Conventi, M. Cooke, B. D. Cooper, A. M. Cooper-Sarkar, T. Cornelissen, M. Corradi, F. Corriveau, A. Corso-Radu, A. Cortes-Gonzalez, G. Cortiana, G. Costa, M. J. Costa, D. Costanzo, D. Côté, G. Cottin, G. Cowan, B. E. Cox, K. Cranmer, G. Cree, S. Crépé-Renaudin, F. Crescioli, W. A. Cribbs, M. Crispin Ortuzar, M. Cristinziani, V. Croft, G. Crosetti, T. Cuhadar Donszelmann, J. Cummings, M. Curatolo, C. Cuthbert, H. Czirr, P. Czodrowski, S. D’Auria, M. D’Onofrio, M. J. Da Cunha Sargedas De Sousa, C. Da Via, W. Dabrowski, A. Dafinca, T. Dai, O. Dale, F. Dallaire, C. Dallapiccola, M. Dam, J. R. Dandoy, N. P. Dang, A. C. Daniells, M. Danninger, M. Dano Hoffmann, V. Dao, G. Darbo, S. Darmora, J. Dassoulas, A. Dattagupta, W. Davey, C. David, T. Davidek, E. Davies, M. Davies, P. Davison, Y. Davygora, E. Dawe, I. Dawson, R. K. Daya-Ishmukhametova, K. De, R. de Asmundis, S. De Castro, S. De Cecco, N. De Groot, P. de Jong, H. De la Torre, F. De Lorenzi, L. De Nooij, D. De Pedis, A. De Salvo, U. De Sanctis, A. De Santo, J. B. De Vivie De Regie, W. J. Dearnaley, R. Debbe, C. Debenedetti, D. V. Dedovich, I. Deigaard, J. Del Peso, T. Del Prete, D. Delgove, F. Deliot, C. M. Delitzsch, M. Deliyergiyev, A. Dell’Acqua, L. Dell’Asta, M. Dell’Orso, M. Della Pietra, D. della Volpe, M. Delmastro, P. A. Delsart, C. Deluca, D. A. DeMarco, S. Demers, M. Demichev, A. Demilly, S. P. Denisov, D. Derendarz, J. E. Derkaoui, F. Derue, P. Dervan, K. Desch, C. Deterre, P. O. Deviveiros, A. Dewhurst, S. Dhaliwal, A. Di Ciaccio, L. Di Ciaccio, A. Di Domenico, C. Di Donato, A. Di Girolamo, B. Di Girolamo, A. Di Mattia, B. Di Micco, R. Di Nardo, A. Di Simone, R. Di Sipio, D. Di Valentino, C. Diaconu, M. Diamond, F. A. Dias, M. A. Diaz, E. B. Diehl, J. Dietrich, S. Diglio, A. Dimitrievska, J. Dingfelder, P. Dita, S. Dita, F. Dittus, F. Djama, T. Djobava, J. I. Djuvsland, M. A. B. do Vale, D. Dobos, M. Dobre, C. Doglioni, T. Dohmae, J. Dolejsi, Z. Dolezal, B. A. Dolgoshein, M. Donadelli, S. Donati, P. Dondero, J. Donini, J. Dopke, A. Doria, M. T. Dova, A. T. Doyle, E. Drechsler, M. Dris, E. Dubreuil, E. Duchovni, G. Duckeck, O. A. Ducu, D. Duda, A. Dudarev, L. Duflot, L. Duguid, M. Dührssen, M. Dunford, H. Duran Yildiz, M. Düren, A. Durglishvili, D. Duschinger, M. Dyndal, C. Eckardt, K. M. Ecker, R. C. Edgar, W. Edson, N. C. Edwards, W. Ehrenfeld, T. Eifert, G. Eigen, K. Einsweiler, T. Ekelof, M. El Kacimi, M. Ellert, S. Elles, F. Ellinghaus, A. A. Elliot, N. Ellis, J. Elmsheuser, M. Elsing, D. Emeliyanov, Y. Enari, O. C. Endner, M. Endo, J. Erdmann, A. Ereditato, G. Ernis, J. Ernst, M. Ernst, S. Errede, E. Ertel, M. Escalier, H. Esch, C. Escobar, B. Esposito, A. I. Etienvre, E. Etzion, H. Evans, A. Ezhilov, L. Fabbri, G. Facini, R. M. Fakhrutdinov, S. Falciano, R. J. Falla, J. Faltova, Y. Fang, M. Fanti, A. Farbin, A. Farilla, T. Farooque, S. Farrell, S. M. Farrington, P. Farthouat, F. Fassi, P. Fassnacht, D. Fassouliotis, M. Faucci Giannelli, A. Favareto, L. Fayard, P. Federic, O. L. Fedin, W. Fedorko, S. Feigl, L. Feligioni, C. Feng, E. J. Feng, H. Feng, A. B. Fenyuk, L. Feremenga, P. Fernandez Martinez, S. Fernandez Perez, J. Ferrando, A. Ferrari, P. Ferrari, R. Ferrari, D. E. Ferreira de Lima, A. Ferrer, D. Ferrere, C. Ferretti, A. Ferretto Parodi, M. Fiascaris, F. Fiedler, A. Filipčič, M. Filipuzzi, F. Filthaut, M. Fincke-Keeler, K. D. Finelli, M. C. N. Fiolhais, L. Fiorini, A. Firan, A. Fischer, C. Fischer, J. Fischer, W. C. Fisher, E. A. Fitzgerald, I. Fleck, P. Fleischmann, S. Fleischmann, G. T. Fletcher, G. Fletcher, R. R. M. Fletcher, T. Flick, A. Floderus, L. R. Flores Castillo, M. J. Flowerdew, A. Formica, A. Forti, D. Fournier, H. Fox, S. Fracchia, P. Francavilla, M. Franchini, D. Francis, L. Franconi, M. Franklin, M. Frate, M. Fraternali, D. Freeborn, S. T. French, F. Friedrich, D. Froidevaux, J. A. Frost, C. Fukunaga, E. Fullana Torregrosa, B. G. Fulsom, J. Fuster, C. Gabaldon, O. Gabizon, A. Gabrielli, A. Gabrielli, S. Gadatsch, S. Gadomski, G. Gagliardi, P. Gagnon, C. Galea, B. Galhardo, E. J. Gallas, B. J. Gallop, P. Gallus, G. Galster, K. K. Gan, J. Gao, Y. Gao, Y. S. Gao, F. M. Garay Walls, F. Garberson, C. García, J. E. García Navarro, M. Garcia-Sciveres, R. W. Gardner, N. Garelli, V. Garonne, C. Gatti, A. Gaudiello, G. Gaudio, B. Gaur, L. Gauthier, P. Gauzzi, I. L. Gavrilenko, C. Gay, G. Gaycken, E. N. Gazis, P. Ge, Z. Gecse, C. N. P. Gee, D. A. A. Geerts, Ch. Geich-Gimbel, M. P. Geisler, C. Gemme, M. H. Genest, S. Gentile, M. George, S. George, D. Gerbaudo, A. Gershon, H. Ghazlane, B. Giacobbe, S. Giagu, V. Giangiobbe, P. Giannetti, B. Gibbard, S. M. Gibson, M. Gilchriese, T. P. S. Gillam, D. Gillberg, G. Gilles, D. M. Gingrich, N. Giokaris, M. P. Giordani, F. M. Giorgi, F. M. Giorgi, P. F. Giraud, P. Giromini, D. Giugni, C. Giuliani, M. Giulini, B. K. Gjelsten, S. Gkaitatzis, I. Gkialas, E. L. Gkougkousis, L. K. Gladilin, C. Glasman, J. Glatzer, P. C. F. Glaysher, A. Glazov, M. Goblirsch-Kolb, J. R. Goddard, J. Godlewski, S. Goldfarb, T. Golling, D. Golubkov, A. Gomes, R. Gonçalo, J. Goncalves Pinto Firmino Da Costa, L. Gonella, S. González de la Hoz, G. Gonzalez Parra, S. Gonzalez-Sevilla, L. Goossens, P. A. Gorbounov, H. A. Gordon, I. Gorelov, B. Gorini, E. Gorini, A. Gorišek, E. Gornicki, A. T. Goshaw, C. Gössling, M. I. Gostkin, D. Goujdami, A. G. Goussiou, N. Govender, E. Gozani, H. M. X. Grabas, L. Graber, I. Grabowska-Bold, P. Grafström, K-J. Grahn, J. Gramling, E. Gramstad, S. Grancagnolo, V. Grassi, V. Gratchev, H. M. Gray, E. Graziani, Z. D. Greenwood, K. Gregersen, I. M. Gregor, P. Grenier, J. Griffiths, A. A. Grillo, K. Grimm, S. Grinstein, Ph. Gris, J.-F. Grivaz, J. P. Grohs, A. Grohsjean, E. Gross, J. Grosse-Knetter, G. C. Grossi, Z. J. Grout, L. Guan, J. Guenther, F. Guescini, D. Guest, O. Gueta, E. Guido, T. Guillemin, S. Guindon, U. Gul, C. Gumpert, J. Guo, S. Gupta, G. Gustavino, P. Gutierrez, N. G. Gutierrez Ortiz, C. Gutschow, C. Guyot, C. Gwenlan, C. B. Gwilliam, A. Haas, C. Haber, H. K. Hadavand, N. Haddad, P. Haefner, S. Hageböck, Z. Hajduk, H. Hakobyan, M. Haleem, J. Haley, D. Hall, G. Halladjian, G. D. Hallewell, K. Hamacher, P. Hamal, K. Hamano, M. Hamer, A. Hamilton, G. N. Hamity, P. G. Hamnett, L. Han, K. Hanagaki, K. Hanawa, M. Hance, P. Hanke, R. Hanna, J. B. Hansen, J. D. Hansen, M. C. Hansen, P. H. Hansen, K. Hara, A. S. Hard, T. Harenberg, F. Hariri, S. Harkusha, R. D. Harrington, P. F. Harrison, F. Hartjes, M. Hasegawa, S. Hasegawa, Y. Hasegawa, A. Hasib, S. Hassani, S. Haug, R. Hauser, L. Hauswald, M. Havranek, C. M. Hawkes, R. J. Hawkings, A. D. Hawkins, T. Hayashi, D. Hayden, C. P. Hays, J. M. Hays, H. S. Hayward, S. J. Haywood, S. J. Head, T. Heck, V. Hedberg, L. Heelan, S. Heim, T. Heim, B. Heinemann, L. Heinrich, J. Hejbal, L. Helary, S. Hellman, D. Hellmich, C. Helsens, J. Henderson, R. C. W. Henderson, Y. Heng, C. Hengler, A. Henrichs, A. M. Henriques Correia, S. Henrot-Versille, G. H. Herbert, Y. Hernández Jiménez, R. Herrberg-Schubert, G. Herten, R. Hertenberger, L. Hervas, G. G. Hesketh, N. P. Hessey, J. W. Hetherly, R. Hickling, E. Higón-Rodriguez, E. Hill, J. C. Hill, K. H. Hiller, S. J. Hillier, I. Hinchliffe, E. Hines, R. R. Hinman, M. Hirose, D. Hirschbuehl, J. Hobbs, N. Hod, M. C. Hodgkinson, P. Hodgson, A. Hoecker, M. R. Hoeferkamp, F. Hoenig, M. Hohlfeld, D. Hohn, T. R. Holmes, M. Homann, T. M. Hong, L. Hooft van Huysduynen, W. H. Hopkins, Y. Horii, A. J. Horton, J-Y. Hostachy, S. Hou, A. Hoummada, J. Howard, J. Howarth, M. Hrabovsky, I. Hristova, J. Hrivnac, T. Hryn’ova, A. Hrynevich, C. Hsu, P. J. Hsu, S.-C. Hsu, D. Hu, Q. Hu, X. Hu, Y. Huang, Z. Hubacek, F. Hubaut, F. Huegging, T. B. Huffman, E. W. Hughes, G. Hughes, M. Huhtinen, T. A. Hülsing, N. Huseynov, J. Huston, J. Huth, G. Iacobucci, G. Iakovidis, I. Ibragimov, L. Iconomidou-Fayard, E. Ideal, Z. Idrissi, P. Iengo, O. Igonkina, T. Iizawa, Y. Ikegami, K. Ikematsu, M. Ikeno, Y. Ilchenko, D. Iliadis, N. Ilic, Y. Inamaru, T. Ince, P. Ioannou, M. Iodice, K. Iordanidou, V. Ippolito, A. Irles Quiles, C. Isaksson, M. Ishino, M. Ishitsuka, R. Ishmukhametov, C. Issever, S. Istin, J. M. Iturbe Ponce, R. Iuppa, J. Ivarsson, W. Iwanski, H. Iwasaki, J. M. Izen, V. Izzo, S. Jabbar, B. Jackson, M. Jackson, P. Jackson, M. R. Jaekel, V. Jain, K. Jakobs, S. Jakobsen, T. Jakoubek, J. Jakubek, D. O. Jamin, D. K. Jana, E. Jansen, R. Jansky, J. Janssen, M. Janus, G. Jarlskog, N. Javadov, T. Javůrek, L. Jeanty, J. Jejelava, G.-Y. Jeng, D. Jennens, P. Jenni, J. Jentzsch, C. Jeske, S. Jézéquel, H. Ji, J. Jia, Y. Jiang, S. Jiggins, J. Jimenez Pena, S. Jin, A. Jinaru, O. Jinnouchi, M. D. Joergensen, P. Johansson, K. A. Johns, K. Jon-And, G. Jones, R. W. L. Jones, T. J. Jones, J. Jongmanns, P. M. Jorge, K. D. Joshi, J. Jovicevic, X. Ju, C. A. Jung, P. Jussel, A. Juste Rozas, M. Kaci, A. Kaczmarska, M. Kado, H. Kagan, M. Kagan, S. J. Kahn, E. Kajomovitz, C. W. Kalderon, S. Kama, A. Kamenshchikov, N. Kanaya, M. Kaneda, S. Kaneti, V. A. Kantserov, J. Kanzaki, B. Kaplan, A. Kapliy, D. Kar, K. Karakostas, A. Karamaoun, N. Karastathis, M. J. Kareem, M. Karnevskiy, S. N. Karpov, Z. M. Karpova, K. Karthik, V. Kartvelishvili, A. N. Karyukhin, L. Kashif, R. D. Kass, A. Kastanas, Y. Kataoka, A. Katre, J. Katzy, K. Kawagoe, T. Kawamoto, G. Kawamura, S. Kazama, V. F. Kazanin, M. Y. Kazarinov, R. Keeler, R. Kehoe, J. S. Keller, J. J. Kempster, H. Keoshkerian, O. Kepka, B. P. Kerševan, S. Kersten, R. A. Keyes, F. Khalil-zada, H. Khandanyan, A. Khanov, A. G. Kharlamov, T. J. Khoo, V. Khovanskiy, E. Khramov, J. Khubua, H. Y. Kim, H. Kim, S. H. Kim, Y. K. Kim, N. Kimura, O. M. Kind, B. T. King, M. King, S. B. King, J. Kirk, A. E. Kiryunin, T. Kishimoto, D. Kisielewska, F. Kiss, K. Kiuchi, O. Kivernyk, E. Kladiva, M. H. Klein, M. Klein, U. Klein, K. Kleinknecht, P. Klimek, A. Klimentov, R. Klingenberg, J. A. Klinger, T. Klioutchnikova, E.-E. Kluge, P. Kluit, S. Kluth, E. Kneringer, E. B. F. G. Knoops, A. Knue, A. Kobayashi, D. Kobayashi, T. Kobayashi, M. Kobel, M. Kocian, P. Kodys, T. Koffas, E. Koffeman, L. A. Kogan, S. Kohlmann, Z. Kohout, T. Kohriki, T. Koi, H. Kolanoski, I. Koletsou, A. A. Komar, Y. Komori, T. Kondo, N. Kondrashova, K. Köneke, A. C. König, S. König, T. Kono, R. Konoplich, N. Konstantinidis, R. Kopeliansky, S. Koperny, L. Köpke, A. K. Kopp, K. Korcyl, K. Kordas, A. Korn, A. A. Korol, I. Korolkov, E. V. Korolkova, O. Kortner, S. Kortner, T. Kosek, V. V. Kostyukhin, V. M. Kotov, A. Kotwal, A. Kourkoumeli-Charalampidi, C. Kourkoumelis, V. Kouskoura, A. Koutsman, R. Kowalewski, T. Z. Kowalski, W. Kozanecki, A. S. Kozhin, V. A. Kramarenko, G. Kramberger, D. Krasnopevtsev, M. W. Krasny, A. Krasznahorkay, J. K. Kraus, A. Kravchenko, S. Kreiss, M. Kretz, J. Kretzschmar, K. Kreutzfeldt, P. Krieger, K. Krizka, K. Kroeninger, H. Kroha, J. Kroll, J. Kroseberg, J. Krstic, U. Kruchonak, H. Krüger, N. Krumnack, Z. V. Krumshteyn, A. Kruse, M. C. Kruse, M. Kruskal, T. Kubota, H. Kucuk, S. Kuday, S. Kuehn, A. Kugel, F. Kuger, A. Kuhl, T. Kuhl, V. Kukhtin, Y. Kulchitsky, S. Kuleshov, M. Kuna, T. Kunigo, A. Kupco, H. Kurashige, Y. A. Kurochkin, R. Kurumida, V. Kus, E. S. Kuwertz, M. Kuze, J. Kvita, T. Kwan, D. Kyriazopoulos, A. La Rosa, J. L. La Rosa Navarro, L. La Rotonda, C. Lacasta, F. Lacava, J. Lacey, H. Lacker, D. Lacour, V. R. Lacuesta, E. Ladygin, R. Lafaye, B. Laforge, T. Lagouri, S. Lai, L. Lambourne, S. Lammers, C. L. Lampen, W. Lampl, E. Lançon, U. Landgraf, M. P. J. Landon, V. S. Lang, J. C. Lange, A. J. Lankford, F. Lanni, K. Lantzsch, S. Laplace, C. Lapoire, J. F. Laporte, T. Lari, F. Lasagni Manghi, M. Lassnig, P. Laurelli, W. Lavrijsen, A. T. Law, P. Laycock, T. Lazovich, O. Le Dortz, E. Le Guirriec, E. Le Menedeu, M. LeBlanc, T. LeCompte, F. Ledroit-Guillon, C. A. Lee, S. C. Lee, L. Lee, G. Lefebvre, M. Lefebvre, F. Legger, C. Leggett, A. Lehan, G. Lehmann Miotto, X. Lei, W. A. Leight, A. Leisos, A. G. Leister, M. A. L. Leite, R. Leitner, D. Lellouch, B. Lemmer, K. J. C. Leney, T. Lenz, B. Lenzi, R. Leone, S. Leone, C. Leonidopoulos, S. Leontsinis, C. Leroy, C. G. Lester, M. Levchenko, J. Levêque, D. Levin, L. J. Levinson, M. Levy, A. Lewis, A. M. Leyko, M. Leyton, B. Li, H. Li, H. L. Li, L. Li, L. Li, S. Li, Y. Li, Z. Liang, H. Liao, B. Liberti, A. Liblong, P. Lichard, K. Lie, J. Liebal, W. Liebig, C. Limbach, A. Limosani, S. C. Lin, T. H. Lin, F. Linde, B. E. Lindquist, J. T. Linnemann, E. Lipeles, A. Lipniacka, M. Lisovyi, T. M. Liss, D. Lissauer, A. Lister, A. M. Litke, B. Liu, D. Liu, H. Liu, J. Liu, J. B. Liu, K. Liu, L. Liu, M. Liu, M. Liu, Y. Liu, M. Livan, A. Lleres, J. Llorente Merino, S. L. Lloyd, F. Lo Sterzo, E. Lobodzinska, P. Loch, W. S. Lockman, F. K. Loebinger, A. E. Loevschall-Jensen, A. Loginov, T. Lohse, K. Lohwasser, M. Lokajicek, B. A. Long, J. D. Long, R. E. Long, K. A. Looper, L. Lopes, D. Lopez Mateos, B. Lopez Paredes, I. Lopez Paz, J. Lorenz, N. Lorenzo Martinez, M. Losada, P. Loscutoff, P. J. Lösel, X. Lou, A. Lounis, J. Love, P. A. Love, N. Lu, H. J. Lubatti, C. Luci, A. Lucotte, F. Luehring, W. Lukas, L. Luminari, O. Lundberg, B. Lund-Jensen, D. Lynn, R. Lysak, E. Lytken, H. Ma, L. L. Ma, G. Maccarrone, A. Macchiolo, C. M. Macdonald, J. Machado Miguens, D. Macina, D. Madaffari, R. Madar, H. J. Maddocks, W. F. Mader, A. Madsen, S. Maeland, T. Maeno, A. Maevskiy, E. Magradze, K. Mahboubi, J. Mahlstedt, C. Maiani, C. Maidantchik, A. A. Maier, T. Maier, A. Maio, S. Majewski, Y. Makida, N. Makovec, B. Malaescu, Pa. Malecki, V. P. Maleev, F. Malek, U. Mallik, D. Malon, C. Malone, S. Maltezos, V. M. Malyshev, S. Malyukov, J. Mamuzic, G. Mancini, B. Mandelli, L. Mandelli, I. Mandić, R. Mandrysch, J. Maneira, A. Manfredini, L. Manhaes de Andrade Filho, J. Manjarres Ramos, A. Mann, P. M. Manning, A. Manousakis-Katsikakis, B. Mansoulie, R. Mantifel, M. Mantoani, L. Mapelli, L. March, G. Marchiori, M. Marcisovsky, C. P. Marino, M. Marjanovic, D. E. Marley, F. Marroquim, S. P. Marsden, Z. Marshall, L. F. Marti, S. Marti-Garcia, B. Martin, T. A. Martin, V. J. Martin, B. Martin dit Latour, M. Martinez, S. Martin-Haugh, V. S. Martoiu, A. C. Martyniuk, M. Marx, F. Marzano, A. Marzin, L. Masetti, T. Mashimo, R. Mashinistov, J. Masik, A. L. Maslennikov, I. Massa, L. Massa, N. Massol, P. Mastrandrea, A. Mastroberardino, T. Masubuchi, P. Mättig, J. Mattmann, J. Maurer, S. J. Maxfield, D. A. Maximov, R. Mazini, S. M. Mazza, L. Mazzaferro, G. Mc Goldrick, S. P. Mc Kee, A. McCarn, R. L. McCarthy, T. G. McCarthy, N. A. McCubbin, K. W. McFarlane, J. A. Mcfayden, G. Mchedlidze, S. J. McMahon, R. A. McPherson, M. Medinnis, S. Meehan, S. Mehlhase, A. Mehta, K. Meier, C. Meineck, B. Meirose, B. R. Mellado Garcia, F. Meloni, A. Mengarelli, S. Menke, E. Meoni, K. M. Mercurio, S. Mergelmeyer, P. Mermod, L. Merola, C. Meroni, F. S. Merritt, A. Messina, J. Metcalfe, A. S. Mete, C. Meyer, C. Meyer, J-P. Meyer, J. Meyer, R. P. Middleton, S. Miglioranzi, L. Mijović, G. Mikenberg, M. Mikestikova, M. Mikuž, M. Milesi, A. Milic, D. W. Miller, C. Mills, A. Milov, D. A. Milstead, A. A. Minaenko, Y. Minami, I. A. Minashvili, A. I. Mincer, B. Mindur, M. Mineev, Y. Ming, L. M. Mir, T. Mitani, J. Mitrevski, V. A. Mitsou, A. Miucci, P. S. Miyagawa, J. U. Mjörnmark, T. Moa, K. Mochizuki, S. Mohapatra, W. Mohr, S. Molander, R. Moles-Valls, K. Mönig, C. Monini, J. Monk, E. Monnier, J. Montejo Berlingen, F. Monticelli, S. Monzani, R. W. Moore, N. Morange, D. Moreno, M. Moreno Llácer, P. Morettini, M. Morgenstern, M. Morii, M. Morinaga, V. Morisbak, S. Moritz, A. K. Morley, G. Mornacchi, J. D. Morris, S. S. Mortensen, A. Morton, L. Morvaj, M. Mosidze, J. Moss, K. Motohashi, R. Mount, E. Mountricha, S. V. Mouraviev, E. J. W. Moyse, S. Muanza, R. D. Mudd, F. Mueller, J. Mueller, K. Mueller, R. S. P. Mueller, T. Mueller, D. Muenstermann, P. Mullen, G. A. Mullier, Y. Munwes, J. A. Murillo Quijada, W. J. Murray, H. Musheghyan, E. Musto, A. G. Myagkov, M. Myska, O. Nackenhorst, J. Nadal, K. Nagai, R. Nagai, Y. Nagai, K. Nagano, A. Nagarkar, Y. Nagasaka, K. Nagata, M. Nagel, E. Nagy, A. M. Nairz, Y. Nakahama, K. Nakamura, T. Nakamura, I. Nakano, H. Namasivayam, R. F. Naranjo Garcia, R. Narayan, T. Naumann, G. Navarro, R. Nayyar, H. A. Neal, P. Yu. Nechaeva, T. J. Neep, P. D. Nef, A. Negri, M. Negrini, S. Nektarijevic, C. Nellist, A. Nelson, S. Nemecek, P. Nemethy, A. A. Nepomuceno, M. Nessi, M. S. Neubauer, M. Neumann, R. M. Neves, P. Nevski, P. R. Newman, D. H. Nguyen, R. B. Nickerson, R. Nicolaidou, B. Nicquevert, J. Nielsen, N. Nikiforou, A. Nikiforov, V. Nikolaenko, I. Nikolic-Audit, K. Nikolopoulos, J. K. Nilsen, P. Nilsson, Y. Ninomiya, A. Nisati, R. Nisius, T. Nobe, M. Nomachi, I. Nomidis, T. Nooney, S. Norberg, M. Nordberg, O. Novgorodova, S. Nowak, M. Nozaki, L. Nozka, K. Ntekas, G. Nunes Hanninger, T. Nunnemann, E. Nurse, F. Nuti, B. J. O’Brien, F. O’grady, D. C. O’Neil, V. O’Shea, F. G. Oakham, H. Oberlack, T. Obermann, J. Ocariz, A. Ochi, I. Ochoa, J. P. Ochoa-Ricoux, S. Oda, S. Odaka, H. Ogren, A. Oh, S. H. Oh, C. C. Ohm, H. Ohman, H. Oide, W. Okamura, H. Okawa, Y. Okumura, T. Okuyama, A. Olariu, S. A. Olivares Pino, D. Oliveira Damazio, E. Oliver Garcia, A. Olszewski, J. Olszowska, A. Onofre, P. U. E. Onyisi, C. J. Oram, M. J. Oreglia, Y. Oren, D. Orestano, N. Orlando, C. Oropeza Barrera, R. S. Orr, B. Osculati, R. Ospanov, G. Otero y Garzon, H. Otono, M. Ouchrif, E. A. Ouellette, F. Ould-Saada, A. Ouraou, K. P. Oussoren, Q. Ouyang, A. Ovcharova, M. Owen, R. E. Owen, V. E. Ozcan, N. Ozturk, K. Pachal, A. Pacheco Pages, C. Padilla Aranda, M. Pagáčová, S. Pagan Griso, E. Paganis, C. Pahl, F. Paige, P. Pais, K. Pajchel, G. Palacino, S. Palestini, M. Palka, D. Pallin, A. Palma, Y. B. Pan, E. Panagiotopoulou, C. E. Pandini, J. G. Panduro Vazquez, P. Pani, S. Panitkin, D. Pantea, L. Paolozzi, Th. D. Papadopoulou, K. Papageorgiou, A. Paramonov, D. Paredes Hernandez, M. A. Parker, K. A. Parker, F. Parodi, J. A. Parsons, U. Parzefall, E. Pasqualucci, S. Passaggio, F. Pastore, Fr. Pastore, G. Pásztor, S. Pataraia, N. D. Patel, J. R. Pater, T. Pauly, J. Pearce, B. Pearson, L. E. Pedersen, M. Pedersen, S. Pedraza Lopez, R. Pedro, S. V. Peleganchuk, D. Pelikan, H. Peng, B. Penning, J. Penwell, D. V. Perepelitsa, E. Perez Codina, M. T. Pérez García-Estañ, L. Perini, H. Pernegger, S. Perrella, R. Peschke, V. D. Peshekhonov, K. Peters, R. F. Y. Peters, B. A. Petersen, T. C. Petersen, E. Petit, A. Petridis, C. Petridou, E. Petrolo, F. Petrucci, N. E. Pettersson, R. Pezoa, P. W. Phillips, G. Piacquadio, E. Pianori, A. Picazio, E. Piccaro, M. Piccinini, M. A. Pickering, R. Piegaia, D. T. Pignotti, J. E. Pilcher, A. D. Pilkington, J. Pina, M. Pinamonti, J. L. Pinfold, A. Pingel, B. Pinto, S. Pires, M. Pitt, C. Pizio, L. Plazak, M.-A. Pleier, V. Pleskot, E. Plotnikova, P. Plucinski, D. Pluth, R. Poettgen, L. Poggioli, D. Pohl, G. Polesello, A. Poley, A. Policicchio, R. Polifka, A. Polini, C. S. Pollard, V. Polychronakos, K. Pommès, L. Pontecorvo, B. G. Pope, G. A. Popeneciu, D. S. Popovic, A. Poppleton, S. Pospisil, K. Potamianos, I. N. Potrap, C. J. Potter, C. T. Potter, G. Poulard, J. Poveda, V. Pozdnyakov, P. Pralavorio, A. Pranko, S. Prasad, S. Prell, D. Price, L. E. Price, M. Primavera, S. Prince, M. Proissl, K. Prokofiev, F. Prokoshin, E. Protopapadaki, S. Protopopescu, J. Proudfoot, M. Przybycien, E. Ptacek, D. Puddu, E. Pueschel, D. Puldon, M. Purohit, P. Puzo, J. Qian, G. Qin, Y. Qin, A. Quadt, D. R. Quarrie, W. B. Quayle, M. Queitsch-Maitland, D. Quilty, S. Raddum, V. Radeka, V. Radescu, S. K. Radhakrishnan, P. Radloff, P. Rados, F. Ragusa, G. Rahal, S. Rajagopalan, M. Rammensee, C. Rangel-Smith, F. Rauscher, S. Rave, T. Ravenscroft, M. Raymond, A. L. Read, N. P. Readioff, D. M. Rebuzzi, A. Redelbach, G. Redlinger, R. Reece, K. Reeves, L. Rehnisch, H. Reisin, M. Relich, C. Rembser, H. Ren, A. Renaud, M. Rescigno, S. Resconi, O. L. Rezanova, P. Reznicek, R. Rezvani, R. Richter, S. Richter, E. Richter-Was, O. Ricken, M. Ridel, P. Rieck, C. J. Riegel, J. Rieger, M. Rijssenbeek, A. Rimoldi, L. Rinaldi, B. Ristić, E. Ritsch, I. Riu, F. Rizatdinova, E. Rizvi, S. H. Robertson, A. Robichaud-Veronneau, D. Robinson, J. E. M. Robinson, A. Robson, C. Roda, S. Roe, O. Røhne, S. Rolli, A. Romaniouk, M. Romano, S. M. Romano Saez, E. Romero Adam, N. Rompotis, M. Ronzani, L. Roos, E. Ros, S. Rosati, K. Rosbach, P. Rose, P. L. Rosendahl, O. Rosenthal, V. Rossetti, E. Rossi, L. P. Rossi, R. Rosten, M. Rotaru, I. Roth, J. Rothberg, D. Rousseau, C. R. Royon, A. Rozanov, Y. Rozen, X. Ruan, F. Rubbo, I. Rubinskiy, V. I. Rud, C. Rudolph, M. S. Rudolph, F. Rühr, A. Ruiz-Martinez, Z. Rurikova, N. A. Rusakovich, A. Ruschke, H. L. Russell, J. P. Rutherfoord, N. Ruthmann, Y. F. Ryabov, M. Rybar, G. Rybkin, N. C. Ryder, A. F. Saavedra, G. Sabato, S. Sacerdoti, A. Saddique, H. F-W. Sadrozinski, R. Sadykov, F. Safai Tehrani, M. Saimpert, H. Sakamoto, Y. Sakurai, G. Salamanna, A. Salamon, M. Saleem, D. Salek, P. H. Sales De Bruin, D. Salihagic, A. Salnikov, J. Salt, D. Salvatore, F. Salvatore, A. Salvucci, A. Salzburger, D. Sampsonidis, A. Sanchez, J. Sánchez, V. Sanchez Martinez, H. Sandaker, R. L. Sandbach, H. G. Sander, M. P. Sanders, M. Sandhoff, C. Sandoval, R. Sandstroem, D. P. C. Sankey, M. Sannino, A. Sansoni, C. Santoni, R. Santonico, H. Santos, I. Santoyo Castillo, K. Sapp, A. Sapronov, J. G. Saraiva, B. Sarrazin, O. Sasaki, Y. Sasaki, K. Sato, G. Sauvage, E. Sauvan, G. Savage, P. Savard, C. Sawyer, L. Sawyer, J. Saxon, C. Sbarra, A. Sbrizzi, T. Scanlon, D. A. Scannicchio, M. Scarcella, V. Scarfone, J. Schaarschmidt, P. Schacht, D. Schaefer, R. Schaefer, J. Schaeffer, S. Schaepe, S. Schaetzel, U. Schäfer, A. C. Schaffer, D. Schaile, R. D. Schamberger, V. Scharf, V. A. Schegelsky, D. Scheirich, M. Schernau, C. Schiavi, C. Schillo, M. Schioppa, S. Schlenker, E. Schmidt, K. Schmieden, C. Schmitt, S. Schmitt, S. Schmitt, B. Schneider, Y. J. Schnellbach, U. Schnoor, L. Schoeffel, A. Schoening, B. D. Schoenrock, E. Schopf, A. L. S. Schorlemmer, M. Schott, D. Schouten, J. Schovancova, S. Schramm, M. Schreyer, C. Schroeder, N. Schuh, M. J. Schultens, H.-C. Schultz-Coulon, H. Schulz, M. Schumacher, B. A. Schumm, Ph. Schune, C. Schwanenberger, A. Schwartzman, T. A. Schwarz, Ph. Schwegler, H. Schweiger, Ph. Schwemling, R. Schwienhorst, J. Schwindling, T. Schwindt, F. G. Sciacca, E. Scifo, G. Sciolla, F. Scuri, F. Scutti, J. Searcy, G. Sedov, E. Sedykh, P. Seema, S. C. Seidel, A. Seiden, F. Seifert, J. M. Seixas, G. Sekhniaidze, K. Sekhon, S. J. Sekula, D. M. Seliverstov, N. Semprini-Cesari, C. Serfon, L. Serin, L. Serkin, T. Serre, M. Sessa, R. Seuster, H. Severini, T. Sfiligoj, F. Sforza, A. Sfyrla, E. Shabalina, M. Shamim, L. Y. Shan, R. Shang, J. T. Shank, M. Shapiro, P. B. Shatalov, K. Shaw, S. M. Shaw, A. Shcherbakova, C. Y. Shehu, P. Sherwood, L. Shi, S. Shimizu, C. O. Shimmin, M. Shimojima, M. Shiyakova, A. Shmeleva, D. Shoaleh Saadi, M. J. Shochet, S. Shojaii, S. Shrestha, E. Shulga, M. A. Shupe, S. Shushkevich, P. Sicho, O. Sidiropoulou, D. Sidorov, A. Sidoti, F. Siegert, Dj. Sijacki, J. Silva, Y. Silver, S. B. Silverstein, V. Simak, O. Simard, Lj. Simic, S. Simion, E. Simioni, B. Simmons, D. Simon, R. Simoniello, P. Sinervo, N. B. Sinev, G. Siragusa, A. N. Sisakyan, S. Yu. Sivoklokov, J. Sjölin, T. B. Sjursen, M. B. Skinner, H. P. Skottowe, P. Skubic, M. Slater, T. Slavicek, M. Slawinska, K. Sliwa, V. Smakhtin, B. H. Smart, L. Smestad, S. Yu. Smirnov, Y. Smirnov, L. N. Smirnova, O. Smirnova, M. N. K. Smith, R. W. Smith, M. Smizanska, K. Smolek, A. A. Snesarev, G. Snidero, S. Snyder, R. Sobie, F. Socher, A. Soffer, D. A. Soh, C. A. Solans, M. Solar, J. Solc, E. Yu. Soldatov, U. Soldevila, A. A. Solodkov, A. Soloshenko, O. V. Solovyanov, V. Solovyev, P. Sommer, H. Y. Song, N. Soni, A. Sood, A. Sopczak, B. Sopko, V. Sopko, V. Sorin, D. Sosa, M. Sosebee, C. L. Sotiropoulou, R. Soualah, A. M. Soukharev, D. South, B. C. Sowden, S. Spagnolo, M. Spalla, F. Spanò, W. R. Spearman, F. Spettel, R. Spighi, G. Spigo, L. A. Spiller, M. Spousta, T. Spreitzer, R. D. St. Denis, S. Staerz, J. Stahlman, R. Stamen, S. Stamm, E. Stanecka, C. Stanescu, M. Stanescu-Bellu, M. M. Stanitzki, S. Stapnes, E. A. Starchenko, J. Stark, P. Staroba, P. Starovoitov, R. Staszewski, P. Stavina, P. Steinberg, B. Stelzer, H. J. Stelzer, O. Stelzer-Chilton, H. Stenzel, S. Stern, G. A. Stewart, J. A. Stillings, M. C. Stockton, M. Stoebe, G. Stoicea, P. Stolte, S. Stonjek, A. R. Stradling, A. Straessner, M. E. Stramaglia, J. Strandberg, S. Strandberg, A. Strandlie, E. Strauss, M. Strauss, P. Strizenec, R. Ströhmer, D. M. Strom, R. Stroynowski, A. Strubig, S. A. Stucci, B. Stugu, N. A. Styles, D. Su, J. Su, R. Subramaniam, A. Succurro, Y. Sugaya, C. Suhr, M. Suk, V. V. Sulin, S. Sultansoy, T. Sumida, S. Sun, X. Sun, J. E. Sundermann, K. Suruliz, G. Susinno, M. R. Sutton, S. Suzuki, Y. Suzuki, M. Svatos, S. Swedish, M. Swiatlowski, I. Sykora, T. Sykora, D. Ta, C. Taccini, K. Tackmann, J. Taenzer, A. Taffard, R. Tafirout, N. Taiblum, H. Takai, R. Takashima, H. Takeda, T. Takeshita, Y. Takubo, M. Talby, A. A. Talyshev, J. Y. C. Tam, K. G. Tan, J. Tanaka, R. Tanaka, S. Tanaka, B. B. Tannenwald, N. Tannoury, S. Tapprogge, S. Tarem, F. Tarrade, G. F. Tartarelli, P. Tas, M. Tasevsky, T. Tashiro, E. Tassi, A. Tavares Delgado, Y. Tayalati, F. E. Taylor, G. N. Taylor, W. Taylor, F. A. Teischinger, M. Teixeira Dias Castanheira, P. Teixeira-Dias, K. K. Temming, H. Ten Kate, P. K. Teng, J. J. Teoh, F. Tepel, S. Terada, K. Terashi, J. Terron, S. Terzo, M. Testa, R. J. Teuscher, J. Therhaag, T. Theveneaux-Pelzer, J. P. Thomas, J. Thomas-Wilsker, E. N. Thompson, P. D. Thompson, R. J. Thompson, A. S. Thompson, L. A. Thomsen, E. Thomson, M. Thomson, R. P. Thun, M. J. Tibbetts, R. E. Ticse Torres, V. O. Tikhomirov, Yu. A. Tikhonov, S. Timoshenko, E. Tiouchichine, P. Tipton, S. Tisserant, T. Todorov, S. Todorova-Nova, J. Tojo, S. Tokár, K. Tokushuku, K. Tollefson, E. Tolley, L. Tomlinson, M. Tomoto, L. Tompkins, K. Toms, E. Torrence, H. Torres, E. Torró Pastor, J. Toth, F. Touchard, D. R. Tovey, T. Trefzger, L. Tremblet, A. Tricoli, I. M. Trigger, S. Trincaz-Duvoid, M. F. Tripiana, W. Trischuk, B. Trocmé, C. Troncon, M. Trottier-McDonald, M. Trovatelli, P. True, L. Truong, M. Trzebinski, A. Trzupek, C. Tsarouchas, J. C-L. Tseng, P. V. Tsiareshka, D. Tsionou, G. Tsipolitis, N. Tsirintanis, S. Tsiskaridze, V. Tsiskaridze, E. G. Tskhadadze, I. I. Tsukerman, V. Tsulaia, S. Tsuno, D. Tsybychev, A. Tudorache, V. Tudorache, A. N. Tuna, S. A. Tupputi, S. Turchikhin, D. Turecek, R. Turra, A. J. Turvey, P. M. Tuts, A. Tykhonov, M. Tylmad, M. Tyndel, I. Ueda, R. Ueno, M. Ughetto, M. Ugland, M. Uhlenbrock, F. Ukegawa, G. Unal, A. Undrus, G. Unel, F. C. Ungaro, Y. Unno, C. Unverdorben, J. Urban, P. Urquijo, P. Urrejola, G. Usai, A. Usanova, L. Vacavant, V. Vacek, B. Vachon, C. Valderanis, N. Valencic, S. Valentinetti, A. Valero, L. Valery, S. Valkar, E. Valladolid Gallego, S. Vallecorsa, J. A. Valls Ferrer, W. Van Den Wollenberg, P. C. Van Der Deijl, R. van der Geer, H. van der Graaf, R. Van Der Leeuw, N. van Eldik, P. van Gemmeren, J. Van Nieuwkoop, I. van Vulpen, M. C. van Woerden, M. Vanadia, W. Vandelli, R. Vanguri, A. Vaniachine, F. Vannucci, G. Vardanyan, R. Vari, E. W. Varnes, T. Varol, D. Varouchas, A. Vartapetian, K. E. Varvell, V. I. Vassilakopoulos, F. Vazeille, T. Vazquez Schroeder, J. Veatch, L. M. Veloce, F. Veloso, T. Velz, S. Veneziano, A. Ventura, D. Ventura, M. Venturi, N. Venturi, A. Venturini, V. Vercesi, M. Verducci, W. Verkerke, J. C. Vermeulen, A. Vest, M. C. Vetterli, O. Viazlo, I. Vichou, T. Vickey, O. E. Vickey Boeriu, G. H. A. Viehhauser, S. Viel, R. Vigne, M. Villa, M. Villaplana Perez, E. Vilucchi, M. G. Vincter, V. B. Vinogradov, I. Vivarelli, F. Vives Vaque, S. Vlachos, D. Vladoiu, M. Vlasak, M. Vogel, P. Vokac, G. Volpi, M. Volpi, H. von der Schmitt, H. von Radziewski, E. von Toerne, V. Vorobel, K. Vorobev, M. Vos, R. Voss, J. H. Vossebeld, N. Vranjes, M. Vranjes Milosavljevic, V. Vrba, M. Vreeswijk, R. Vuillermet, I. Vukotic, Z. Vykydal, P. Wagner, W. Wagner, H. Wahlberg, S. Wahrmund, J. Wakabayashi, J. Walder, R. Walker, W. Walkowiak, C. Wang, F. Wang, H. Wang, H. Wang, J. Wang, J. Wang, K. Wang, R. Wang, S. M. Wang, T. Wang, X. Wang, C. Wanotayaroj, A. Warburton, C. P. Ward, D. R. Wardrope, M. Warsinsky, A. Washbrook, C. Wasicki, P. M. Watkins, A. T. Watson, I. J. Watson, M. F. Watson, G. Watts, S. Watts, B. M. Waugh, S. Webb, M. S. Weber, S. W. Weber, J. S. Webster, A. R. Weidberg, B. Weinert, J. Weingarten, C. Weiser, H. Weits, P. S. Wells, T. Wenaus, T. Wengler, S. Wenig, N. Wermes, M. Werner, P. Werner, M. Wessels, J. Wetter, K. Whalen, A. M. Wharton, A. White, M. J. White, R. White, S. White, D. Whiteson, F. J. Wickens, W. Wiedenmann, M. Wielers, P. Wienemann, C. Wiglesworth, L. A. M. Wiik-Fuchs, A. Wildauer, H. G. Wilkens, H. H. Williams, S. Williams, C. Willis, S. Willocq, A. Wilson, J. A. Wilson, I. Wingerter-Seez, F. Winklmeier, B. T. Winter, M. Wittgen, J. Wittkowski, S. J. Wollstadt, M. W. Wolter, H. Wolters, B. K. Wosiek, J. Wotschack, M. J. Woudstra, K. W. Wozniak, M. Wu, M. Wu, S. L. Wu, X. Wu, Y. Wu, T. R. Wyatt, B. M. Wynne, S. Xella, D. Xu, L. Xu, B. Yabsley, S. Yacoob, R. Yakabe, M. Yamada, Y. Yamaguchi, A. Yamamoto, S. Yamamoto, T. Yamanaka, K. Yamauchi, Y. Yamazaki, Z. Yan, H. Yang, H. Yang, Y. Yang, W-M. Yao, Y. Yasu, E. Yatsenko, K. H. Yau Wong, J. Ye, S. Ye, I. Yeletskikh, A. L. Yen, E. Yildirim, K. Yorita, R. Yoshida, K. Yoshihara, C. Young, C. J. S. Young, S. Youssef, D. R. Yu, J. Yu, J. M. Yu, J. Yu, L. Yuan, A. Yurkewicz, I. Yusuff, B. Zabinski, R. Zaidan, A. M. Zaitsev, J. Zalieckas, A. Zaman, S. Zambito, L. Zanello, D. Zanzi, C. Zeitnitz, M. Zeman, A. Zemla, K. Zengel, O. Zenin, T. Ženiš, D. Zerwas, D. Zhang, F. Zhang, H. Zhang, J. Zhang, L. Zhang, R. Zhang, X. Zhang, Z. Zhang, X. Zhao, Y. Zhao, Z. Zhao, A. Zhemchugov, J. Zhong, B. Zhou, C. Zhou, L. Zhou, L. Zhou, N. Zhou, C. G. Zhu, H. Zhu, J. Zhu, Y. Zhu, X. Zhuang, K. Zhukov, A. Zibell, D. Zieminska, N. I. Zimine, C. Zimmermann, S. Zimmermann, Z. Zinonos, M. Zinser, M. Ziolkowski, L. Živković, G. Zobernig, A. Zoccoli, M. zur Nedden, G. Zurzolo, L. Zwalinski, ATLAS Collaboration

**Affiliations:** Department of Physics, University of Adelaide, Adelaide, Australia; Physics Department, SUNY Albany, Albany, NY USA; Department of Physics, University of Alberta, Edmonton, AB Canada; Department of Physics, Ankara University, Ankara, Turkey; Istanbul Aydin University, Istanbul, Turkey; Division of Physics, TOBB University of Economics and Technology, Ankara, Turkey; LAPP, CNRS/IN2P3 and Université Savoie Mont Blanc, Annecy-le-Vieux, France; High Energy Physics Division, Argonne National Laboratory, Argonne, IL USA; Department of Physics, University of Arizona, Tucson, AZ USA; Department of Physics, The University of Texas at Arlington, Arlington, TX USA; Physics Department, University of Athens, Athens, Greece; Physics Department, National Technical University of Athens, Zografou, Greece; Institute of Physics, Azerbaijan Academy of Sciences, Baku, Azerbaijan; Institut de Física d’Altes Energies and Departament de Física de la Universitat Autònoma de Barcelona, Barcelona, Spain; Institute of Physics, University of Belgrade, Belgrade, Serbia; Department for Physics and Technology, University of Bergen, Bergen, Norway; Physics Division, Lawrence Berkeley National Laboratory and University of California, Berkeley, CA USA; Department of Physics, Humboldt University, Berlin, Germany; Albert Einstein Center for Fundamental Physics and Laboratory for High Energy Physics, University of Bern, Bern, Switzerland; School of Physics and Astronomy, University of Birmingham, Birmingham, UK; Department of Physics, Bogazici University, Istanbul, Turkey; Department of Physics Engineering, Gaziantep University, Gaziantep, Turkey; Department of Physics, Dogus University, Gaziantep, Turkey; INFN Sezione di Bologna, Bologna, Italy; Dipartimento di Fisica e Astronomia, Università di Bologna, Bologna, Italy; Physikalisches Institut, University of Bonn, Bonn, Germany; Department of Physics, Boston University, Boston, MA USA; Department of Physics, Brandeis University, Waltham, MA USA; Universidade Federal do Rio De Janeiro COPPE/EE/IF, Rio de Janeiro, Brazil; Electrical Circuits Department, Federal University of Juiz de Fora (UFJF), Juiz de Fora, Brazil; Federal University of Sao Joao del Rei (UFSJ), Sao Joao del Rei, Brazil; Instituto de Fisica, Universidade de Sao Paulo, São Paulo, Brazil; Physics Department, Brookhaven National Laboratory, Upton, NY USA; National Institute of Physics and Nuclear Engineering, Bucharest, Romania; Physics Department, National Institute for Research and Development of Isotopic and Molecular Technologies, Cluj Napoca, Romania; University Politehnica Bucharest, Bucharest, Romania; West University in Timisoara, Timisoara, Romania; Departamento de Física, Universidad de Buenos Aires, Buenos Aires, Argentina; Cavendish Laboratory, University of Cambridge, Cambridge, UK; Department of Physics, Carleton University, Ottawa, ON Canada; CERN, Geneva, Switzerland; Enrico Fermi Institute, University of Chicago, Chicago, IL USA; Departamento de Física, Pontificia Universidad Católica de Chile, Santiago, Chile; Departamento de Física, Universidad Técnica Federico Santa María, Valparaiso, Chile; Institute of High Energy Physics, Chinese Academy of Sciences, Beijing, China; Department of Modern Physics, University of Science and Technology of China, Hefei, Anhui China; Department of Physics, Nanjing University, Nanjing, Jiangsu China; School of Physics, Shandong University, Shandong, China; Shanghai Key Laboratory for Particle Physics and Cosmology, Department of Physics and Astronomy, Shanghai Jiao Tong University, Shanghai, China; Physics Department, Tsinghua University, Beijing, 100084 China; Laboratoire de Physique Corpusculaire, Clermont Université and Université Blaise Pascal and CNRS/IN2P3, Clermont-Ferrand, France; Nevis Laboratory, Columbia University, Irvington, NY USA; Niels Bohr Institute, University of Copenhagen, Copenhagen, Denmark; INFN Gruppo Collegato di Cosenza, Laboratori Nazionali di Frascati, Frascati, Italy; Dipartimento di Fisica, Università della Calabria, Rende, Italy; AGH University of Science and Technology, Faculty of Physics and Applied Computer Science, Kraków, Poland; Marian Smoluchowski Institute of Physics, Jagiellonian University, Kraków, Poland; Institute of Nuclear Physics, Polish Academy of Sciences, Kraków, Poland; Physics Department, Southern Methodist University, Dallas, TX USA; Physics Department, University of Texas at Dallas, Richardson, TX USA; DESY, Hamburg and Zeuthen, Germany; Institut für Experimentelle Physik IV, Technische Universität Dortmund, Dortmund, Germany; Institut für Kern- und Teilchenphysik, Technische Universität Dresden, Dresden, Germany; Department of Physics, Duke University, Durham, NC USA; SUPA-School of Physics and Astronomy, University of Edinburgh, Edinburgh, UK; INFN Laboratori Nazionali di Frascati, Frascati, Italy; Fakultät für Mathematik und Physik, Albert-Ludwigs-Universität, Freiburg, Germany; Section de Physique, Université de Genève, Geneva, Switzerland; INFN Sezione di Genova, Genoa, Italy; Dipartimento di Fisica, Università di Genova, Genoa, Italy; E. Andronikashvili Institute of Physics, Iv. Javakhishvili Tbilisi State University, Tbilisi, Georgia; High Energy Physics Institute, Tbilisi State University, Tbilisi, Georgia; II Physikalisches Institut, Justus-Liebig-Universität Giessen, Giessen, Germany; SUPA-School of Physics and Astronomy, University of Glasgow, Glasgow, UK; II Physikalisches Institut, Georg-August-Universität, Göttingen, Germany; Laboratoire de Physique Subatomique et de Cosmologie, Université Grenoble-Alpes, CNRS/IN2P3, Grenoble, France; Department of Physics, Hampton University, Hampton, VA USA; Laboratory for Particle Physics and Cosmology, Harvard University, Cambridge, MA USA; Kirchhoff-Institut für Physik, Ruprecht-Karls-Universität Heidelberg, Heidelberg, Germany; Physikalisches Institut, Ruprecht-Karls-Universität Heidelberg, Heidelberg, Germany; ZITI Institut für technische Informatik, Ruprecht-Karls-Universität Heidelberg, Mannheim, Germany; Faculty of Applied Information Science, Hiroshima Institute of Technology, Hiroshima, Japan; Department of Physics, The Chinese University of Hong Kong, Shatin, NT Hong Kong; Department of Physics, The University of Hong Kong, Hong Kong, Hong Kong; Department of Physics, The Hong Kong University of Science and Technology, Clear Water Bay, Kowloon, Hong Kong, China; Department of Physics, Indiana University, Bloomington, IN USA; Institut für Astro- und Teilchenphysik, Leopold-Franzens-Universität, Innsbruck, Austria; University of Iowa, Iowa City, IA USA; Department of Physics and Astronomy, Iowa State University, Ames, IA USA; Joint Institute for Nuclear Research, JINR Dubna, Dubna, Russia; KEK, High Energy Accelerator Research Organization, Tsukuba, Japan; Graduate School of Science, Kobe University, Kobe, Japan; Faculty of Science, Kyoto University, Kyoto, Japan; Kyoto University of Education, Kyoto, Japan; Department of Physics, Kyushu University, Fukuoka, Japan; Instituto de Física La Plata, Universidad Nacional de La Plata and CONICET, La Plata, Argentina; Physics Department, Lancaster University, Lancaster, UK; INFN Sezione di Lecce, Lecce, Italy; Dipartimento di Matematica e Fisica, Università del Salento, Lecce, Italy; Oliver Lodge Laboratory, University of Liverpool, Liverpool, UK; Department of Physics, Jožef Stefan Institute and University of Ljubljana, Ljubljana, Slovenia; School of Physics and Astronomy, Queen Mary University of London, London, UK; Department of Physics, Royal Holloway University of London, Surrey, UK; Department of Physics and Astronomy, University College London, London, UK; Louisiana Tech University, Ruston, LA USA; Laboratoire de Physique Nucléaire et de Hautes Energies, UPMC and Université Paris-Diderot and CNRS/IN2P3, Paris, France; Fysiska institutionen, Lunds universitet, Lund, Sweden; Departamento de Fisica Teorica C-15, Universidad Autonoma de Madrid, Madrid, Spain; Institut für Physik, Universität Mainz, Mainz, Germany; School of Physics and Astronomy, University of Manchester, Manchester, UK; CPPM, Aix-Marseille Université and CNRS/IN2P3, Marseille, France; Department of Physics, University of Massachusetts, Amherst, MA USA; Department of Physics, McGill University, Montreal, QC Canada; School of Physics, University of Melbourne, Melbourne, VIC Australia; Department of Physics, The University of Michigan, Ann Arbor, MI USA; Department of Physics and Astronomy, Michigan State University, East Lansing, MI USA; INFN Sezione di Milano, Milan, Italy; Dipartimento di Fisica, Università di Milano, Milan, Italy; B.I. Stepanov Institute of Physics, National Academy of Sciences of Belarus, Minsk, Republic of Belarus; National Scientific and Educational Centre for Particle and High Energy Physics, Minsk, Republic of Belarus; Department of Physics, Massachusetts Institute of Technology, Cambridge, MA USA; Group of Particle Physics, University of Montreal, Montreal, QC Canada; P.N. Lebedev Institute of Physics, Academy of Sciences, Moscow, Russia; Institute for Theoretical and Experimental Physics (ITEP), Moscow, Russia; National Research Nuclear University MEPhI, Moscow, Russia; D.V. Skobeltsyn Institute of Nuclear Physics, M.V. Lomonosov Moscow State University, Moscow, Russia; Fakultät für Physik, Ludwig-Maximilians-Universität München, Munich, Germany; Max-Planck-Institut für Physik (Werner-Heisenberg-Institut), Munich, Germany; Nagasaki Institute of Applied Science, Nagasaki, Japan; Graduate School of Science and Kobayashi-Maskawa Institute, Nagoya University, Nagoya, Japan; INFN Sezione di Napoli, Naples, Italy; Dipartimento di Fisica, Università di Napoli, Naples, Italy; Department of Physics and Astronomy, University of New Mexico, Albuquerque, NM USA; Institute for Mathematics, Astrophysics and Particle Physics, Radboud University Nijmegen/Nikhef, Nijmegen, The Netherlands; Nikhef National Institute for Subatomic Physics and University of Amsterdam, Amsterdam, The Netherlands; Department of Physics, Northern Illinois University, De Kalb, IL USA; Budker Institute of Nuclear Physics, SB RAS, Novosibirsk, Russia; Department of Physics, New York University, New York, NY USA; Ohio State University, Columbus, OH USA; Faculty of Science, Okayama University, Okayama, Japan; Homer L. Dodge Department of Physics and Astronomy, University of Oklahoma, Norman, OK USA; Department of Physics, Oklahoma State University, Stillwater, OK USA; Palacký University, RCPTM, Olomouc, Czech Republic; Center for High Energy Physics, University of Oregon, Eugene, OR USA; LAL, Université Paris-Sud and CNRS/IN2P3, Orsay, France; Graduate School of Science, Osaka University, Osaka, Japan; Department of Physics, University of Oslo, Oslo, Norway; Department of Physics, Oxford University, Oxford, UK; INFN Sezione di Pavia, Pavia, Italy; Dipartimento di Fisica, Università di Pavia, Pavia, Italy; Department of Physics, University of Pennsylvania, Philadelphia, PA USA; National Research Centre “Kurchatov Institute” B.P.Konstantinov, Petersburg Nuclear Physics Institute, St. Petersburg, Russia; INFN Sezione di Pisa, Pisa, Italy; Dipartimento di Fisica E. Fermi, Università di Pisa, Pisa, Italy; Department of Physics and Astronomy, University of Pittsburgh, Pittsburgh, PA USA; Laboratório de Instrumentação e Física Experimental de Partículas-LIP, Lisbon, Portugal; Faculdade de Ciências, Universidade de Lisboa, Lisbon, Portugal; Department of Physics, University of Coimbra, Coimbra, Portugal; Centro de Física Nuclear da Universidade de Lisboa, Lisbon, Portugal; Departamento de Fisica, Universidade do Minho, Braga, Portugal; Departamento de Fisica Teorica y del Cosmos and CAFPE, Universidad de Granada, Granada, Spain; Dep Fisica and CEFITEC of Faculdade de Ciencias e Tecnologia, Universidade Nova de Lisboa, Caparica, Portugal; Institute of Physics, Academy of Sciences of the Czech Republic, Prague, Czech Republic; Czech Technical University in Prague, Prague, Czech Republic; Faculty of Mathematics and Physics, Charles University in Prague, Prague, Czech Republic; State Research Center Institute for High Energy Physics, Protvino, Russia; Particle Physics Department, Rutherford Appleton Laboratory, Didcot, UK; INFN Sezione di Roma, Rome, Italy; Dipartimento di Fisica, Sapienza Università di Roma, Rome, Italy; INFN Sezione di Roma Tor Vergata, Rome, Italy; Dipartimento di Fisica, Università di Roma Tor Vergata, Rome, Italy; INFN Sezione di Roma Tre, Rome, Italy; Dipartimento di Matematica e Fisica, Università Roma Tre, Rome, Italy; Faculté des Sciences Ain Chock, Réseau Universitaire de Physique des Hautes Energies-Université Hassan II, Casablanca, Morocco; Centre National de l’Energie des Sciences Techniques Nucleaires, Rabat, Morocco; Faculté des Sciences Semlalia, Université Cadi Ayyad, LPHEA-Marrakech, Marrakech, Morocco; Faculté des Sciences, Université Mohamed Premier and LPTPM, Oujda, Morocco; Faculté des Sciences, Université Mohammed V-Agdal, Rabat, Morocco; DSM/IRFU (Institut de Recherches sur les Lois Fondamentales de l’Univers), CEA Saclay (Commissariat à l’Energie Atomique et aux Energies Alternatives), Gif-sur-Yvette, France; Santa Cruz Institute for Particle Physics, University of California Santa Cruz, Santa Cruz, CA USA; Department of Physics, University of Washington, Seattle, WA USA; Department of Physics and Astronomy, University of Sheffield, Sheffield, UK; Department of Physics, Shinshu University, Nagano, Japan; Fachbereich Physik, Universität Siegen, Siegen, Germany; Department of Physics, Simon Fraser University, Burnaby, BC Canada; SLAC National Accelerator Laboratory, Stanford, CA USA; Faculty of Mathematics, Physics and Informatics, Comenius University, Bratislava, Slovak Republic; Department of Subnuclear Physics, Institute of Experimental Physics of the Slovak Academy of Sciences, Kosice, Slovak Republic; Department of Physics, University of Cape Town, Cape Town, South Africa; Department of Physics, University of Johannesburg, Johannesburg, South Africa; School of Physics, University of the Witwatersrand, Johannesburg, South Africa; Department of Physics, Stockholm University, Stockholm, Sweden; The Oskar Klein Centre, Stockholm, Sweden; Physics Department, Royal Institute of Technology, Stockholm, Sweden; Departments of Physics and Astronomy and Chemistry, Stony Brook University, Stony Brook, NY USA; Department of Physics and Astronomy, University of Sussex, Brighton, UK; School of Physics, University of Sydney, Sydney, Australia; Institute of Physics, Academia Sinica, Taipei, Taiwan; Department of Physics, Technion: Israel Institute of Technology, Haifa, Israel; Raymond and Beverly Sackler School of Physics and Astronomy, Tel Aviv University, Tel Aviv, Israel; Department of Physics, Aristotle University of Thessaloniki, Thessaloníki, Greece; International Center for Elementary Particle Physics and Department of Physics, The University of Tokyo, Tokyo, Japan; Graduate School of Science and Technology, Tokyo Metropolitan University, Tokyo, Japan; Department of Physics, Tokyo Institute of Technology, Tokyo, Japan; Department of Physics, University of Toronto, Toronto, ON Canada; TRIUMF, Vancouver, BC Canada; Department of Physics and Astronomy, York University, Toronto, ON Canada; Faculty of Pure and Applied Sciences, University of Tsukuba, Tsukuba, Japan; Department of Physics and Astronomy, Tufts University, Medford, MA USA; Centro de Investigaciones, Universidad Antonio Narino, Bogotá, Colombia; Department of Physics and Astronomy, University of California Irvine, Irvine, CA USA; INFN Gruppo Collegato di Udine, Sezione di Trieste, Udine, Italy; ICTP, Trieste, Italy; Dipartimento di Chimica Fisica e Ambiente, Università di Udine, Udine, Italy; Department of Physics, University of Illinois, Urbana, IL USA; Department of Physics and Astronomy, University of Uppsala, Uppsala, Sweden; Instituto de Física Corpuscular (IFIC) and Departamento de Física Atómica, Molecular y Nuclear and Departamento de Ingeniería Electrónica and Instituto de Microelectrónica de Barcelona (IMB-CNM), University of Valencia and CSIC, Valencia, Spain; Department of Physics, University of British Columbia, Vancouver, BC Canada; Department of Physics and Astronomy, University of Victoria, Victoria, BC Canada; Department of Physics, University of Warwick, Coventry, UK; Waseda University, Tokyo, Japan; Department of Particle Physics, The Weizmann Institute of Science, Rehovot, Israel; Department of Physics, University of Wisconsin, Madison, WI USA; Fakultät für Physik und Astronomie, Julius-Maximilians-Universität, Würzburg, Germany; Fachbereich C Physik, Bergische Universität Wuppertal, Wuppertal, Germany; Department of Physics, Yale University, New Haven, CT USA; Yerevan Physics Institute, Yerevan, Armenia; Centre de Calcul de l’Institut National de Physique Nucléaire et de Physique des Particules (IN2P3), Villeurbanne, France

## Abstract

The decays $B_c^+ \rightarrow J/\psi D_s^+$ and $B_c^+ \rightarrow J/\psi D_s^{*+}$ are studied with the ATLAS detector at the LHC using a dataset corresponding to integrated luminosities of 4.9 and 20.6 fb$^{-1}$ of *pp* collisions collected at centre-of-mass energies $\sqrt{s} = 7$ TeV and 8 TeV, respectively. Signal candidates are identified through $J/\psi \rightarrow \mu ^+\mu ^-$ and $D_s^{(*)+}\rightarrow \phi \pi ^+(\gamma /\pi ^0)$ decays. With a two-dimensional likelihood fit involving the $B_c^+$ reconstructed invariant mass and an angle between the $\mu ^+$ and $D_s^+$ candidate momenta in the muon pair rest frame, the yields of $B_c^+ \rightarrow J/\psi D_s^+$ and $B_c^+ \rightarrow J/\psi D_s^{*+}$, and the transverse polarisation fraction in $B_c^+ \rightarrow J/\psi D_s^{*+}$ decay are measured. The transverse polarisation fraction is determined to be $\Gamma _{\pm \pm }(B_c^+ \rightarrow J/\psi D_s^{*+})/\Gamma (B_c^+ \rightarrow J/\psi D_s^{*+}) = 0.38 \pm 0.23 \pm 0.07$, and the derived ratio of the branching fractions of the two modes is $\mathcal {B}_{B_c^+ \rightarrow J/\psi D_s^{*+}}/\mathcal {B}_{B_c^+ \rightarrow J/\psi D_s^+} = 2.8 \,^{+1.2}_{-0.8} \pm 0.3$, where the first error is statistical and the second is systematic. Finally, a sample of $B_c^+\rightarrow J/\psi \pi ^+$ decays is used to derive the ratios of branching fractions $\mathcal {B}_{B_c^+ \rightarrow J/\psi D_s^+}/\mathcal {B}_{B_c^+ \rightarrow J/\psi \pi ^+} = 3.8 \pm 1.1 \pm 0.4 \pm 0.2$ and $\mathcal {B}_{B_c^+ \rightarrow J/\psi D_s^{*+}}/\mathcal {B}_{B_c^+ \rightarrow J/\psi \pi ^+} = 10.4 \pm 3.1 \pm 1.5 \pm 0.6$, where the third error corresponds to the uncertainty of the branching fraction of $D_s^+\rightarrow \phi (K^+K^-)\pi ^+$ decay. The available theoretical predictions are generally consistent with the measurement.

## Introduction

**Fig. 1:**
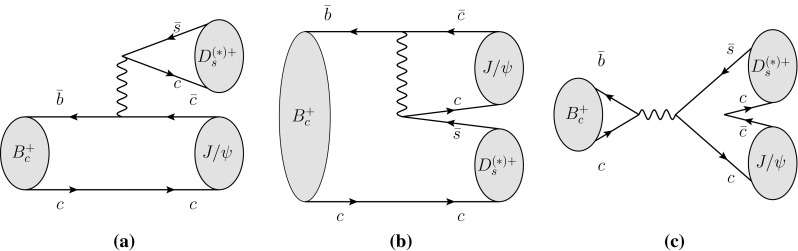
Feynman diagrams for ${B_c^+} \rightarrow {J/\psi } {D_s^{(*)+}}$ decays: **a** colour-favoured spectator, **b** colour-suppressed spectator, and **c** annihilation topology

The $${B_c^+} $$ meson^1^ is the only known weakly decaying particle consisting of two heavy quarks. The ground $$\bar{b}c$$ state was first observed by CDF [1] via its semileptonic decay $${B_c^+} \rightarrow {J/\psi } \ell ^+\nu _\ell $$. An excited $$\bar{b}c$$ state has been observed recently by ATLAS [2] using the $${B_c^+} $$ decay mode $${B_c^+} \rightarrow {J/\psi } \pi ^+$$. The presence of two heavy quarks, each of which can decay weakly, affects theoretical calculations of the decay properties of the $${B_c^+} $$ meson. In the case of $$\bar{b}\rightarrow \bar{c}c\bar{s}$$ processes, decays to charmonium and a $${D_s^+} $$ or a $${D_s^{*+}} $$ meson are predicted to occur via colour-suppressed and colour-favoured spectator diagrams as well as via the weak annihilation diagram (see Fig. 1). The latter, in contrast to decays of other *B* mesons, is not Cabibbo-suppressed and can contribute significantly to the decay amplitudes. The decay properties are addressed in various theoretical calculations [3–9] and can also be compared to the analogous properties in the lighter *B* meson systems such as $${B_d^0} \rightarrow D^{*-}{D_s^{(*)+}} $$ or $$B^+\rightarrow \bar{D}^{*0}{D_s^{(*)+}} $$. The decays $${B_c^+} \rightarrow {J/\psi } {D_s^+} $$ and $${B_c^+} \rightarrow {J/\psi } {D_s^{*+}} $$, which have been observed recently by the LHCb experiment [10], provide a means to test these theoretical predictions.

This paper presents a measurement of the branching fractions of $${B_c^+} \rightarrow {J/\psi } {D_s^+} $$ and $${B_c^+} \rightarrow {J/\psi } {D_s^{*+}} $$ decays, normalised to that of $${B_c^+} \rightarrow {J/\psi } \pi ^+$$ decay, and polarisation in $${B_c^+} \rightarrow {J/\psi } {D_s^{*+}} $$ decay performed with the ATLAS detector [11]. The $${D_s^+} $$ meson is reconstructed via the $${D_s^+} \rightarrow \phi \pi ^+$$ decay with the $$\phi $$ meson decaying into a pair of charged kaons. The $${D_s^{*+}} $$ meson decays into a $${D_s^+} $$ meson and a soft photon or $$\pi ^0$$. Detecting such soft neutral particles is very challenging, thus no attempt to reconstruct them is made in the analysis. The $${J/\psi } $$ meson is reconstructed via its decay into a muon pair.

The measurement presented in this paper allows an independent verification of the results of Ref. [10] with comparable statistical and systematic uncertainties. The following ratios are measured: $${\mathcal {R}}_{{D_s^+}/\pi ^+} = {\mathcal {B}}_{{B_c^+} \rightarrow {J/\psi } {D_s^+}}/{\mathcal {B}}_{{B_c^+} \rightarrow {J/\psi } \pi ^+}$$, $${\mathcal {R}}_{{D_s^{*+}}/\pi ^+} = {\mathcal {B}}_{{B_c^+} \rightarrow {J/\psi } {D_s^{*+}}}/{\mathcal {B}}_{{B_c^+} \rightarrow {J/\psi } \pi ^+}$$, and $${\mathcal {R}}_{{D_s^{*+}}/{D_s^+}} = {\mathcal {B}}_{{B_c^+} \rightarrow {J/\psi } {D_s^{*+}}}/{\mathcal {B}}_{{B_c^+} \rightarrow {J/\psi } {D_s^+}}$$, where $${\mathcal {B}}_{{B_c^+} \rightarrow X}$$ denotes the branching fraction of the $${B_c^+} \rightarrow X$$ decay. The decay $${B_c^+} \rightarrow {J/\psi } {D_s^{*+}} $$ is a transition of a pseudoscalar meson into a pair of vector states and is thus described by the three helicity amplitudes, $$A_{++}$$, $$A_{--}$$, and $$A_{00}$$, where the subscripts correspond to the helicities of $${J/\psi } $$ and $${D_s^{*+}} $$ mesons. The contribution of the $$A_{++}$$ and $$A_{--}$$ amplitudes, referred to as the $$A_{\pm \pm }$$ component, corresponds to the $${J/\psi } $$ and $${D_s^{*+}} $$ transverse polarisation. The fraction of transverse polarisation, $$\Gamma _{\pm \pm }/\Gamma = \Gamma _{\pm \pm } ({B_c^+} \rightarrow {J/\psi } {D_s^{*+}})/\Gamma ({B_c^+} \rightarrow {J/\psi } {D_s^{*+}})$$, is also measured. From a naive prediction by spin counting, one would expect this fraction to be 2 / 3, while calculations [8, 9] predict values of 0.41–0.48.

This analysis is based on a combined sample of *pp* collision data collected by the ATLAS experiment at the LHC at centre-of-mass energies $\sqrt{s} = 7$ TeV and 8 TeV corresponding to integrated luminosities of 4.9 and 20.6 fb$^{-1}$, respectively.

## The ATLAS detector, trigger selection and Monte Carlo samples

ATLAS is a general-purpose detector consisting of several subsystems including the inner detector (ID), calorimeters and the muon spectrometer (MS). Muon reconstruction makes use of both the ID and the MS. The ID comprises three types of detectors: a silicon pixel detector, a silicon microstrip semiconductor tracker (SCT) and a transition radiation tracker. The ID provides a pseudorapidity^2^ coverage up to $|\eta | = 2.5$. Muons pass through the calorimeters and reach the MS if their transverse momentum, ${p_{\text {T}}}$, is above approximately 3 GeV.^3^ Muon candidates are formed either from a stand-alone MS track matched to an ID track or, in case the MS stand-alone track is not reconstructed, from an ID track extrapolated to the MS and matched to track segments in the MS. Candidates of the latter type are referred to as segment-tagged muons while the former are called combined muons. Muon track parameters are taken from the ID measurement alone in this analysis, since the precision of the measured track parameters for muons in the ${p_{\text {T}}}$ range of interest is dominated by the ID track reconstruction.

The ATLAS trigger system consists of a hardware-based Level-1 trigger and a two-stage high level trigger (HLT). At Level-1, the muon trigger uses dedicated MS chambers to search for patterns of hits satisfying different ${p_{\text {T}}}$ thresholds. The region-of-interest around these hit patterns then serves as a seed for the HLT muon reconstruction, in which dedicated algorithms are used to incorporate information from both the MS and the ID, achieving a position and momentum resolution close to that provided by the offline muon reconstruction. Muons are efficiently triggered in the pseudorapidity range $|\eta | < 2.4$.

Triggers based on single-muon, dimuon, and three-muon signatures are used to select $${J/\psi } \rightarrow \mu ^+\mu ^-$$ decays for the analysis. The third muon can be produced in the $${B_c^+} $$ signal events in semileptonic decays of the two other heavy-flavour hadrons. The majority of events are collected by dimuon triggers requiring a vertex of two oppositely charged muons with an invariant mass between 2.5 and 4.3 GeV. During the data taking, the $${p_{\text {T}}} $$ threshold for muons in these triggers was either 4 or 6 GeV. Single-muon triggers additionally increase the acceptance for asymmetric $${J/\psi } $$ decays where one muon has $${p_{\text {T}}} < 4$$ GeV. Finally, three-muon triggers had a $${p_{\text {T}}} $$ threshold of 4 GeV, thus enhancing the acceptance during the periods of high luminosity when the $${p_{\text {T}}} $$ threshold for at least one muon in the dimuon triggers was 6 GeV.

Monte Carlo (MC) simulation is used for the event selection criteria optimisation and the calculation of the acceptance for the considered $${B_c^+} $$ decay modes. The MC samples of the $${B_c^+} $$ decays were generated with Pythia 6.4 [12] along with a dedicated extension for the $${B_c^+} $$ production based on calculations from Refs. [13–16]. The decays of $${B_c^+} $$ are then simulated with EvtGen [17]. The generated events were passed through a full simulation of the detector using the ATLAS simulation framework [18] based on Geant 4 [19, 20] and processed with the same reconstruction algorithms as were used for the data.

## Reconstruction and event selection

The ${J/\psi }$ candidates are reconstructed from pairs of oppositely charged muons. At least one of the two muons is required to be a combined muon. Each pair is fitted to a common vertex [21]. The quality of the vertex fit must satisfy $\chi ^2/{\mathrm {ndf}} < 15$, where the ${\mathrm {ndf}}$ stands for the number of degrees of freedom. The candidates in the invariant mass window $2800\,\mathrm{MeV} < m(\mu ^+\mu ^-) < 3400$ MeV are retained.

For the $${D_s^+} \rightarrow \phi (K^+K^-)\pi ^+$$ reconstruction, tracks of particles with opposite charges are assigned kaon mass hypotheses and combined in pairs to form $$\phi $$ candidates. An additional track is assigned a pion mass and combined with the $$\phi $$ candidate to form a $${D_s^+} $$ candidate. To ensure good momentum resolution, all three tracks are required to have at least two hits in the silicon pixel detector and at least six hits in the SCT. Only three-track combinations successfully fitted to a common vertex with $$\chi ^2/{\mathrm {ndf}} < 8$$ are kept. The $$\phi $$ candidate invariant mass, $$m(K^+K^-)$$, and the $${D_s^+} $$ candidate invariant mass, $$m(K^+K^-\pi ^+)$$, are calculated using the track momenta refitted to the common vertex. Only candidates with $$m(K^+K^-)$$ within $$\pm 7$$ MeV around the $$\phi $$ mass, $$m_{\phi } = 1019.461$$ MeV [22], and with $$1930\ \mathrm{MeV} < m(K^+K^-\pi ^+) < 2010$$ MeV are retained.

The $${B_c^+} \rightarrow {J/\psi } {D_s^+} $$ candidates are built by combining the five tracks of the $${J/\psi } $$ and $${D_s^+} $$ candidates. The $${J/\psi } $$ meson decays instantly at the same point as the $${B_c^+} $$ does (secondary vertex) while the $${D_s^+} $$ lives long enough to form a displaced tertiary vertex. Therefore the five-track combinations are refitted assuming this cascade topology [21]. The invariant mass of the muon pair is constrained to the $${J/\psi } $$ mass, $$m_{{J/\psi }} = 3096.916$$ MeV [22]. The three $${D_s^+} $$ daughter tracks are constrained to a tertiary vertex and their invariant mass is fixed to the mass of $${D_s^+} $$, $$m_{{D_s^+}} = 1968.30$$ MeV [22]. The combined momentum of the refitted $${D_s^+} $$ decay tracks is constrained to point to the dimuon vertex. The quality of the cascade fit must satisfy $$\chi ^2/{\mathrm {ndf}} < 3$$.

The $${B_c^+} $$ meson is reconstructed within the kinematic range $${p_{\text {T}}} ({B_c^+}) > 15$$ GeV and $$|\eta ({B_c^+})| < 2.0$$, where the detector acceptance is high and depends weakly on $${p_{\text {T}}} ({B_c^+})$$ and $$\eta ({B_c^+})$$.

The refitted tracks of the $${D_s^+} $$ daughter hadrons are required to have $$|\eta | < 2.5$$ and $${p_{\text {T}}} > 1$$ GeV, while the muons must have $$|\eta | < 2.3$$ and $${p_{\text {T}}} > 3$$ GeV. To further discriminate the sample of $${D_s^+} $$ candidates from a large combinatorial background, the following requirements are applied:

- $\cos \theta ^*(\pi ) < 0.8$, where $\theta ^*(\pi )$ is the angle between the pion momentum in the $K^+K^-\pi ^+$ rest frame and the $K^+K^-\pi ^+$ combined momentum in the laboratory frame;
- $$|\cos ^3\theta ^\prime (K)| > 0.15$$, where $$\theta ^\prime (K)$$ is the angle between one of the kaons and the pion in the $$K^+K^-$$ rest frame. The decay of the pseudoscalar $${D_s^+} $$ meson to the $$\phi $$ (vector) plus $$\pi $$ (pseudoscalar) final state results in an alignment of the spin of the $$\phi $$ meson perpendicularly to the direction of motion of the $$\phi $$ relative to $${D_s^+} $$. Consequently, the distribution of $$\cos \theta ^\prime (K)$$ follows a $$\cos ^2\theta ^\prime (K)$$ shape, implying a uniform distribution for $$\cos ^3\theta ^\prime (K)$$. In contrast, the $$\cos \theta ^\prime (K)$$ distribution of the combinatorial background is uniform and its $$\cos ^3\theta ^\prime (K)$$ distribution peaks at zero. The cut suppresses the background significantly while reducing the signal by 15 %.

**Fig. 2:**
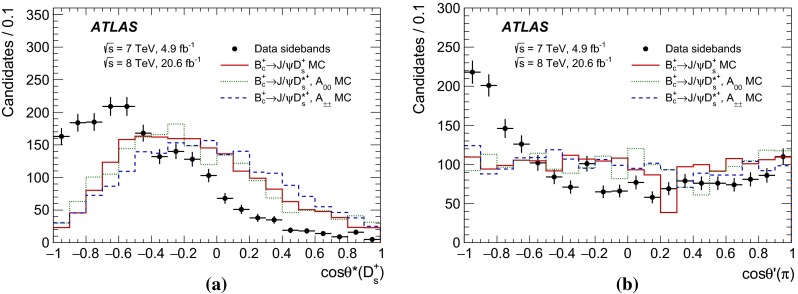
Distributions of **a** $\cos \theta ^*({D_s^+})$ and **b** $\cos \theta ^\prime (\pi )$, where $\theta ^*({D_s^+})$ and $\theta ^\prime (\pi )$ are two angular variables defined in Sect. 3. The distributions are shown for data sidebands (*black dots*) and MC simulation of ${B_c^+} \rightarrow {J/\psi } {D_s^+}$ signal (*red solid line*) and $A_{00}$ (*green dotted line*) and $A_{\pm \pm }$ (*blue dashed line*) components of ${B_c^+} \rightarrow {J/\psi } {D_s^{*+}}$ signal. The distributions are obtained after applying all selection criteria except the ones on the plotted variable. The MC distributions are normalised to data

The $${B_c^+} $$ candidate is required to point back to a primary vertex such that $$d_0^\mathrm{PV}({B_c^+}) < 0.1$$ mm and $$z_0^\mathrm{PV}({B_c^+})\sin \theta ({B_c^+}) < 0.5$$ mm, where $$d_0^\mathrm{PV}$$ and $$z_0^\mathrm{PV}$$ are respectively the transverse and longitudinal impact parameters with respect to the primary vertex. All primary vertices in the event are considered. If there is more than one primary vertex satisfying these requirements ($$\sim $$0.5 % events both in data and MC simulation), the one with the largest sum of squared transverse momenta of the tracks originating from it is chosen.

The transverse decay length^4^ of the $${B_c^+} $$ candidate is required to satisfy $$L_{xy}({B_c^+}) > 0.1$$ mm. The transverse decay length of the $${D_s^+} $$ measured from the $${B_c^+} $$ vertex must be $$L_{xy}({D_s^+}) > 0.15$$ mm. In order to remove fake candidates, both $$L_{xy}({B_c^+})$$ and $$L_{xy}({D_s^+})$$ are required not to exceed 10 mm.

Taking into account the characteristic hard fragmentation of *b*-quarks, a requirement $${p_{\text {T}}} ({B_c^+})/\sum {p_{\text {T}}} (\mathrm {trk}) > 0.1$$ is applied, where the sum in the denominator is taken over all tracks originating from the primary vertex (tracks of the $${B_c^+} $$ candidate are included in the sum if they are associated with the primary vertex). The requirement reduces a sizeable fraction of combinatorial background while having almost no effect on the signal.

The following angular selection requirements are introduced to further suppress the combinatorial background:

- $$\cos \theta ^*({D_s^+}) > -0.8$$, where $$\theta ^*({D_s^+})$$ is the angle between the $${D_s^+} $$ candidate momentum in the rest frame of the $${B_c^+} $$ candidate, and the $${B_c^+} $$ candidate line of flight in the laboratory frame. The distribution of $$\cos \theta ^*({D_s^+})$$ is uniform for the decays of pseudoscalar $${B_c^+} $$ meson before any kinematic selection while it tends to increase for negative values of $$\cos \theta ^*({D_s^+})$$ for the background.
- $\cos \theta ^\prime (\pi ) > -0.8$, where $\theta ^\prime (\pi )$ is the angle between the ${J/\psi }$ candidate momentum and the pion momentum in the $K^+K^-\pi ^+$ rest frame. Its distribution is nearly uniform for the signal processes but peaks towards $-1$ for the background.

Distributions of these two variables after applying all other selection requirements described in this section are shown in Fig. 2. They are shown for the simulated signal samples, as well as for sidebands of the mass spectrum in data, defined as the regions $5640\ \mathrm{MeV} < m({J/\psi } {D_s^+}) < 5900$ MeV (left sideband) and $6360\ \mathrm{MeV} < m({J/\psi } {D_s^+}) < 6760$ MeV (right sideband). A dip in the $\cos \theta ^\prime (\pi )$ distribution for the ${B_c^+} \rightarrow {J/\psi } {D_s^+}$ signal is caused by rejection of ${B_s^0} \rightarrow {J/\psi } \phi$ candidates discussed below.

Various possible contributions of partially reconstructed $$B \rightarrow {J/\psi } X$$ decays were studied. The only significant one was found from the $${B_s^0} \rightarrow {J/\psi } \phi $$ decay process. This contribution arises when the combination of the tracks from a true $${B_s^0} \rightarrow {J/\psi } (\mu ^+\mu ^-)\phi (K^+K^-)$$ decay with a fifth random track results in a fake $${B_c^+} \rightarrow {J/\psi } (\mu ^+\mu ^-){D_s^+} (K^+K^-\pi ^+)$$ candidate. For each reconstructed $${B_c^+} $$ candidate, an additional vertex fit is performed. The two muon tracks and the two kaon tracks are fitted to a common vertex, where the kaon tracks are assumed to be from $$\phi \rightarrow K^+K^-$$ and the muon pair is constrained to have the nominal $${J/\psi } $$ mass. The mass of the $${B_s^0} $$ candidate, $$m(\mu ^+\mu ^-K^+K^-)$$, is then calculated from the refitted track parameters. Candidates with $$5340\ \mathrm{MeV} < m(\mu ^+\mu ^-K^+K^-) < 5400$$ MeV are rejected. This requirement suppresses the bulk of the $${B_s^0} $$ events while rejecting only $$\sim $$4 % of the signal.

After applying the selection requirements described above, 1547 ${J/\psi } {D_s^+}$ candidates are selected in the mass range 5640–6760 MeV.

## ${B_c^+} \rightarrow {J/\psi } {D_s^{(*)+}}$ candidate fit

**Fig. 3:**
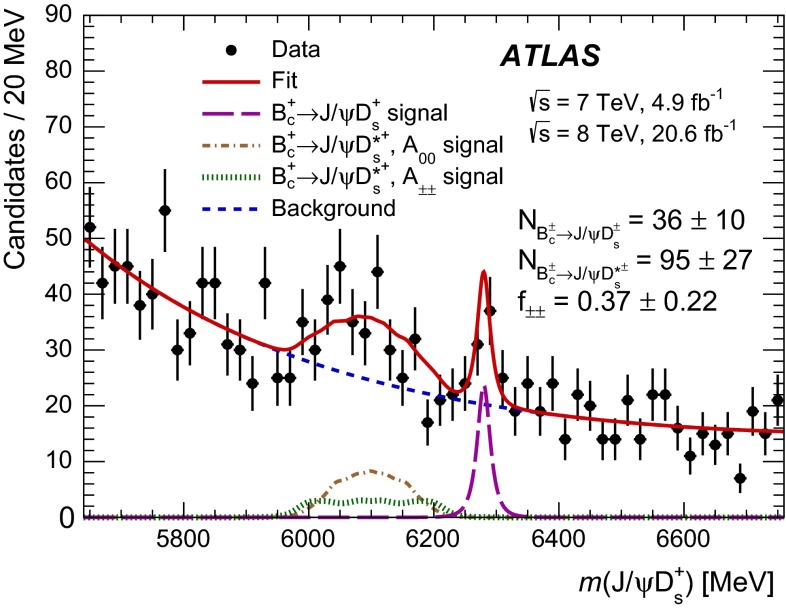
The mass distribution for the selected ${J/\psi } {D_s^+}$ candidates. The *red solid line* represents the projection of the fit to the model described in the text. The contribution of the ${B_c^+} \rightarrow {J/\psi } {D_s^+}$ decay is shown with the *magenta long-dashed line*; the *brown dash-dot* and *green dotted lines* show the ${B_c^+} \rightarrow {J/\psi } {D_s^{*+}}$ $A_{00}$ and $A_{\pm \pm }$ component contributions, respectively; the *blue dashed line* shows the background model. The uncertainties of the listed fit result values are statistical only

The mass distribution of the selected $${B_c^+} \rightarrow {J/\psi } {D_s^{(*)+}} $$ candidates is shown in Fig. 3. The peak near the $${B_c^+} $$ mass, $$m_{{B_c^+}} = 6275.6$$ MeV [22], is attributed to the signal of $${B_c^+} \rightarrow {J/\psi } {D_s^+} $$ decay while a wider structure between 5900 and 6200 MeV corresponds to $${B_c^+} \rightarrow {J/\psi } {D_s^{*+}} $$ with subsequent $${D_s^{*+}} \rightarrow {D_s^+} \gamma $$ or $${D_s^{*+}} \rightarrow {D_s^+} \pi ^0$$ decays where the neutral particle is not reconstructed.

**Fig. 4:**
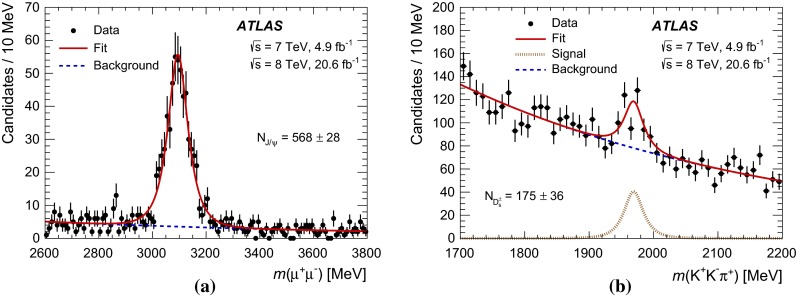
Mass distribution of the **a** $${J/\psi } $$ and **b** $${D_s^+} $$ candidates after the full $${B_c^+} \rightarrow {J/\psi } {D_s^{(*)+}} $$ selection (without mass constraints in the cascade fit) in the mass window of the $${B_c^+} $$ candidate $$5900\ \mathrm{MeV} < m({J/\psi } {D_s^+}) < 6400$$ MeV. The spectra are fitted with a sum of an exponential and a modified Gaussian function. The uncertainties of the shown $${J/\psi } $$ and $${D_s^+} $$ yields are statistical only

Mass distributions of the $${J/\psi } $$ and $${D_s^+} $$ candidates corresponding to the $${J/\psi } {D_s^+} $$ mass region of the observed $${B_c^+} \rightarrow {J/\psi } {D_s^{(*)+}} $$ signals are shown in Fig. 4. To obtain these plots, the $${B_c^+} $$ candidates are built without the mass constraints in the cascade fit, with the mass of the candidate calculated as $$m({J/\psi } {D_s^+}) = m(\mu ^+\mu ^-K^+K^-\pi ^+) - m(\mu ^+\mu ^-) + m_{{J/\psi }} - m(K^+K^-\pi ^+) + m_{{D_s^+}}$$, where $$m_{{J/\psi }}$$ and $$m_{{D_s^+}}$$ are the nominal masses of the respective particles. The mass of the $${B_c^+} $$ candidate is required to be $$5900\ \mathrm{MeV} < m({J/\psi } {D_s^+}) < 6400$$ MeV while the mass windows for the corresponding intermediate resonances are widened to the plotting ranges. The $${J/\psi } $$ and $${D_s^+} $$ mass distributions are fitted with a sum of an exponential function describing the background and a modified Gaussian function [23, 24] describing the corresponding signal peak. The modified Gaussian function is defined as

1 $$\begin{aligned} \mathrm {Gauss}^\mathrm {mod} \sim \exp \left( -\frac{x^{1+\frac{1}{1+x/2}}}{2}\right) , \end{aligned}$$

where $x = |m_0 - m|/\sigma$ with the mean mass $m_0$ and width $\sigma$ being free parameters. The fitted masses of ${J/\psi }$ ($3095.1\pm 2.4$ MeV) and $D_s$ ($1969.0\pm 4.1$ MeV) agree with their nominal masses, the widths are consistent with those in the simulated samples, and the signal yields are found to be $N_{{J/\psi }} = 568 \pm 28$ and $N_{D_s^\pm } = 175 \pm 36$.

The information about the helicity in $${B_c^+} \rightarrow {J/\psi } {D_s^{*+}} $$ decay is encoded both in the mass distribution of the $${J/\psi } {D_s^+} $$ system and in the distribution of the helicity angle, $$\theta ^\prime (\mu ^+)$$, which is defined in the rest frame of the muon pair as the angle between the $$\mu ^+$$ and the $${D_s^+} $$ candidate momenta. Thus, a two-dimensional extended unbinned maximum-likelihood fit of the $$m({J/\psi } {D_s^+})$$ and $$|\cos \theta ^\prime (\mu ^+)|$$ distributions is performed. The $$A_{++}$$ and $$A_{--}$$ helicity amplitude contributions are described by the same mass and angular shapes because of the parity symmetry of the $${J/\psi } $$ and $${D_s^{*+}} $$ decays. This is confirmed by the MC simulation. Thus these components are treated together as the $$A_{\pm \pm }$$ component, while the shape of the $$A_{00}$$ component is different and is therefore treated separately. A simultaneous fit to the mass and angular distributions significantly improves the sensitivity to the contributions of the helicity amplitudes in $${B_c^+} \rightarrow {J/\psi } {D_s^{*+}} $$ decay with respect to a one-dimensional mass fit.

Four two-dimensional probability density functions (PDFs) are defined to describe the ${B_c^+} \rightarrow {J/\psi } {D_s^+}$ signal, the $A_{\pm \pm }$ and $A_{00}$ components of the ${B_c^+} \rightarrow {J/\psi } {D_s^{*+}}$ signal, and the background. The signal PDFs are factorised into mass and angular components. The effect of correlations between their mass and angular shapes is found to be small and is accounted for as a systematic uncertainty.

The mass distribution of the ${B_c^+} \rightarrow {J/\psi } {D_s^+}$ signal is described by a modified Gaussian function. For the ${B_c^+} \rightarrow {J/\psi } {D_s^{*+}}$ signal components, the mass shape templates obtained from the simulation with the kernel estimation technique [25] are used. The branching fractions of ${D_s^{*+}} \rightarrow {D_s^+} \pi ^0$ and ${D_s^{*+}} \rightarrow {D_s^+} \gamma$ decays for the simulation are set to the world average values [22]. The position of the templates along the mass axis is varied in the fit simultaneously with the position of the ${B_c^+} \rightarrow {J/\psi } {D_s^+}$ signal peak. The background mass shape is described with a two-parameter exponential function, $\exp \left[ a\cdot m({J/\psi } {D_s^+}) + b\cdot m({J/\psi } {D_s^+})^2\right]$.

**Fig. 5:**
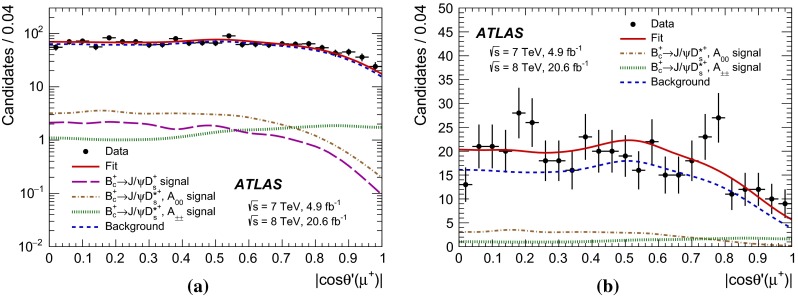
The projection of the likelihood fit on the variable $$|\cos \theta ^\prime (\mu ^+)|$$, where the helicity angle $$\theta ^\prime (\mu ^+)$$ is the angle between the $$\mu ^+$$ and $${D_s^+} $$ candidate momenta in the rest frame of the muon pair from $${J/\psi } $$ decay, for **a** the full selected $${J/\psi } {D_s^+} $$ candidate dataset and **b** a subset of the candidates in a mass range $$5950\ \mathrm{MeV} < m({J/\psi } {D_s^+}) < 6250$$ MeV corresponding to the observed signal of $${B_c^+} \rightarrow {J/\psi } {D_s^{*+}} $$ decay. The *red solid line* represents the full fit projection. The contribution of the $${B_c^+} \rightarrow {J/\psi } {D_s^+} $$ decay is shown with the *magenta long-dashed line* (it is not drawn in **b** because this contribution vanishes in that mass range); the *brown dash-dot* and *green dotted lines* show the $${B_c^+} \rightarrow {J/\psi } {D_s^{*+}} $$ $$A_{00}$$ and $$A_{\pm \pm }$$ component contributions, respectively; the *blue dashed line* shows the background model

To describe the $|\cos \theta ^\prime (\mu ^+)|$ shapes, templates from the kernel estimation are used. The templates for the signal angular PDFs are extracted from the simulated samples. Although their shapes are calculable analytically, using the templates allows the fit to account for detector effects. The background angular description is based on the $|\cos \theta ^\prime (\mu ^+)|$ shape of the candidates in the sidebands of ${J/\psi } {D_s^+}$ mass spectra. Two templates are produced from the angular distributions of the candidates in the left and right mass sidebands as defined in Sect. 3. The angular PDF for the background is defined as a conditional PDF of $|\cos \theta ^\prime (\mu ^+)|$ given the per-candidate $m({J/\psi } {D_s^+})$. For the candidates in the lower half of the left sideband (5640–5770 MeV), the template from the left sideband is used. Similarly, the template from the right sideband is used for the upper half of the right sideband (6560–6760 MeV). For the candidates in the middle part of the mass spectrum (5770–6560 MeV), a linear interpolation between the two templates is used.

**Table 1:** Parameters of the ${B_c^+} \rightarrow {J/\psi } {D_s^{(*)+}}$ signals obtained with the unbinned extended maximum-likelihood fit. The width parameter of the modified Gaussian function is fixed to the MC value. Only statistical uncertainties are shown. No acceptance corrections are applied to the signal yields

Parameter	Value
$m_{{B_c^+} \rightarrow {J/\psi } {D_s^+}}$ (MeV)	$6279.9 \pm 3.5$
$N_{{B_c^+} \rightarrow {J/\psi } {D_s^+}}$	$36 \pm 10$
$N_{{B_c^+} \rightarrow {J/\psi } {D_s^{*+}}}$	$95 \pm 27$
${f_{\pm \pm }}$	$0.37 \pm 0.22$

The fit has seven free parameters: the mass of the $${B_c^+} $$ meson, $$m_{{B_c^+} \rightarrow {J/\psi } {D_s^+}}$$; the relative contribution of the $$A_{\pm \pm }$$ component to the total $${B_c^+} \rightarrow {J/\psi } {D_s^{*+}} $$ decay rate in the selected sample, $${f_{\pm \pm }}$$; the two parameters of the exponential background; the yields of the two signal modes, $$N_{{B_c^+} \rightarrow {J/\psi } {D_s^+}}$$ and $$N_{{B_c^+} \rightarrow {J/\psi } {D_s^{*+}}}$$, and the background yield. The width of the modified Gaussian function, $$\sigma _{{B_c^+} \rightarrow {J/\psi } {D_s^+}}$$, is fixed to the value obtained from the fit to the simulated signal, $$\sigma _{{B_c^+} \rightarrow {J/\psi } {D_s^+}} = 9.95$$ MeV. Leaving this parameter free in the data fit results in the value $$7.9 \pm 3.0$$ MeV, consistent with the simulation in the range of statistical uncertainty.

It was checked that the fit procedure provides unbiased values and correct statistical uncertainties for the extracted parameters using pseudo-experiments. The values of the relevant parameters obtained from the fit are given in Table 1. The fitted $${B_c^+} $$ mass agrees with the world average value [22]. The mass and angular projections of the fit on the selected $${J/\psi } {D_s^+} $$ candidate dataset are also shown in Figs. 3 and 5a, respectively. In order to illustrate the effect of the angular part of the fit in separating the helicity amplitudes, the $$|\cos \theta ^\prime (\mu ^+)|$$ projection for the subset of candidates with the masses $$5950\ \mathrm{MeV} < m({J/\psi } {D_s^+}) < 6250$$ MeV corresponding to the region of the observed $${B_c^+} \rightarrow {J/\psi } {D_s^{*+}} $$ signal is shown in Fig. 5b.

The statistical significance for the observed $${B_c^+} $$ signal estimated from toy MC studies is 4.9 standard deviations.

## ${B_c^+} \rightarrow {J/\psi } \pi ^+$ candidate reconstruction and fit

${B_c^+} \rightarrow {J/\psi } \pi ^+$ candidates are reconstructed by fitting a common vertex of a pion candidate track and the two muons from a ${J/\psi }$ candidate, selected as described in Sect. 3. For the pion candidate, tracks identified as muons are vetoed in order to suppress the substantial background from ${B_c^+} \rightarrow {J/\psi } \mu ^+\nu _\mu X$ decays. The invariant mass of the two muons in the vertex fit is constrained to the ${J/\psi }$ nominal mass. The quality of the fit must satisfy $\chi ^2/{\mathrm {ndf}} < 3$. The following selection requirements applied to the ${B_c^+} \rightarrow {J/\psi } \pi ^+$ candidates are analogous to those for ${B_c^+} \rightarrow {J/\psi } {D_s^+}$ candidates described in Sect. 3: the candidates must be within the kinematic range ${p_{\text {T}}} ({B_c^+}) > 15$ GeV, $|\eta ({B_c^+})| < 2.0$; the refitted values of the transverse momenta and pseudorapidities of the muons are required to satisfy ${p_{\text {T}}} (\mu ^\pm ) > 3$ GeV, $|\eta (\mu ^\pm )|<2.3$; the same requirements on pointing to the primary vertex and the ratio ${p_{\text {T}}} ({B_c^+})/\sum {p_{\text {T}}} (\mathrm {trk})$ are applied. The refitted pion track kinematics must satisfy ${p_{\text {T}}} (\pi ^+) > 5$ GeV and $|\eta (\pi ^+)|<2.5$. The transverse decay length is required to be $L_{xy}({B_c^+}) > 0.2$ mm, and not to exceed 10 mm.

To further suppress combinatorial background, the following selection is applied:

- $$\cos \theta ^*(\pi ) > -0.8$$, where $$\theta ^*(\pi )$$ is the angle between the pion momentum in the $$\mu ^+\mu ^-\pi ^+$$ rest frame and the $${B_c^+} $$ candidate line of flight in laboratory frame. This angular variable behaviour for the signal and the background is the same as that of $$\cos \theta ^*({D_s^+})$$ used for $${J/\psi } {D_s^+} $$ candidates selection.
- $|\cos \theta ^\prime (\mu ^+)| < 0.8$, where $\theta ^\prime (\mu ^+)$ is the angle between the $\mu ^+$ and $\pi ^+$ momenta in the muon pair rest frame. The signal distribution follows a $\sin ^2\theta ^\prime (\mu ^+)$ shape, while the background is flat.

**Fig. 6:**
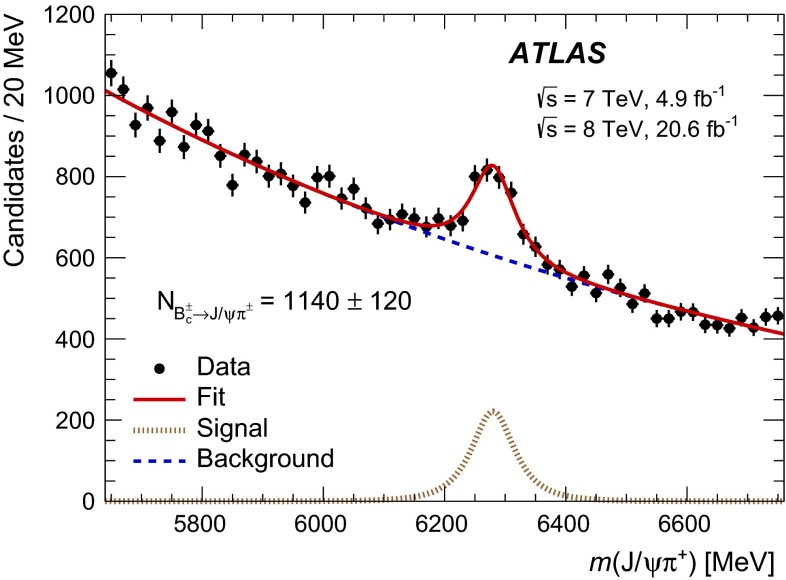
The mass distribution for the selected ${B_c^+} \rightarrow {J/\psi } \pi ^+$ candidates. The *red solid line* represents the result of the fit to the model described in the text. The *brown dotted* and *blue dashed lines* show the signal and background component projections, respectively. The uncertainty of the shown signal yield is statistical only

After applying the above-mentioned requirements, 38542 $${J/\psi } \pi ^+$$ candidates are selected in the mass range 5640–6760 MeV. Figure 6 shows the mass distribution of the selected candidates. An extended unbinned maximum-likelihood fit of the mass spectrum is performed to evaluate the $${B_c^+} \rightarrow {J/\psi } \pi ^+$$ signal yield. The signal contribution is described with the modified Gaussian function while an exponential function is used for the background. The $${B_c^+} $$ mass, $$m_{{B_c^+} \rightarrow {J/\psi } \pi ^+}$$, the width of the modified Gaussian function, $$\sigma _{{B_c^+} \rightarrow {J/\psi } \pi ^+}$$, the yields of the signal, $$N_{{B_c^+} \rightarrow {J/\psi } \pi ^+}$$, and the background, and the slope of the exponential background are free parameters of the fit. The fit results are summarised in Table 2, and the fit projection is also shown in Fig. 6. The extracted $${B_c^+} $$ mass value is consistent with the world average [22], and the signal peak width agrees with the simulation (37.4 MeV).

**Table 2:** Signal parameters of the ${J/\psi } \pi ^+$ mass distribution obtained with the unbinned extended maximum-likelihood fit. Only statistical uncertainties are shown. No acceptance corrections are applied to the signal yields

Parameter	Value
$m_{{B_c^+} \rightarrow {J/\psi } \pi ^+}$ (MeV)	$6279.9 \pm 3.9$
$\sigma _{{B_c^+} \rightarrow {J/\psi } \pi ^+}$ (MeV)	$33.9 \pm 4.2$
$N_{{B_c^+} \rightarrow {J/\psi } \pi ^+}$	$1140 \pm 120$

## Branching fractions and polarisation measurement

The ratios of the branching fractions ${\mathcal {R}}_{{D_s^+}/\pi ^+}$ and ${\mathcal {R}}_{{D_s^{*+}}/\pi ^+}$ are calculated as

2 $$\begin{aligned} {\mathcal {R}}_{{D_s^{(*)+}}/\pi ^+}= & {} \frac{{\mathcal {B}}_{{B_c^+} \rightarrow {J/\psi } {D_s^{(*)+}}}}{{\mathcal {B}}_{{B_c^+} \rightarrow {J/\psi } \pi ^+}} = \frac{1}{{\mathcal {B}}_{{D_s^+} \rightarrow \phi (K^+K^-)\pi ^+}} \nonumber \\&\times \frac{{\mathcal {A}}_{{B_c^+} \rightarrow {J/\psi } \pi ^+}}{{\mathcal {A}}_{{B_c^+} \rightarrow {J/\psi } {D_s^{(*)+}}}} \times \frac{N_{{B_c^+} \rightarrow {J/\psi } {D_s^{(*)+}}}}{N_{{B_c^+} \rightarrow {J/\psi } \pi ^+}}, \end{aligned}$$

where $${\mathcal {A}}_{{B_c^+} \rightarrow X}$$ and $$N_{{B_c^+} \rightarrow X}$$ are the total acceptance and the yield of the corresponding mode. For $${\mathcal {B}}_{{D_s^+} \rightarrow \phi (K^+K^-)\pi ^+}$$, the CLEO measurement [26] of the partial $${D_s^+} \rightarrow K^+K^-\pi ^+$$ branching fractions, with a kaon-pair mass within various intervals around the nominal $$\phi $$ meson mass, is used. An interpolation between the partial branching fractions, measured for $$\pm 5$$ and $$\pm 10$$ MeV intervals, using a relativistic Breit–Wigner shape of the resonance yields the value $$(1.85 \pm 0.11)$$% for the $$\pm 7$$ MeV interval which is used in the analysis. The effect of admixture of other $${D_s^+} $$ decay modes with $$(K^+K^-\pi ^+)$$ final state which are not present in the MC simulation is studied separately and accounted for as a systematic uncertainty.

The acceptance for the ${B_c^+} \rightarrow {J/\psi } {D_s^{*+}}$ decay mode is different for the $A_{\pm \pm }$ and $A_{00}$ components, thus the full acceptance for the mode is

3 $$\begin{aligned}&{\mathcal {A}}_{{B_c^+} \rightarrow {J/\psi } {D_s^{*+}}} \nonumber \\&\quad = \left( \frac{{f_{\pm \pm }}}{{\mathcal {A}}_{{B_c^+} \rightarrow {J/\psi } {D_s^{*+}},A_{\pm \pm }}} + \frac{1-{f_{\pm \pm }}}{{\mathcal {A}}_{{B_c^+} \rightarrow {J/\psi } {D_s^{*+}},A_{00}}}\right) ^{-1}, \end{aligned}$$

where the subscripts indicate the helicity state and ${f_{\pm \pm }}$ is the value extracted from the fit (Table 1). The acceptances are determined from the simulation and shown in Table 3.

**Table 3:** The acceptance ${\mathcal {A}}_{{B_c^+} \rightarrow X}$ for all decay modes studied. Only uncertainties due to MC statistics are shown

Mode	${\mathcal {A}}_{{B_c^+} \rightarrow X}$ (%)
${B_c^+} \rightarrow {J/\psi } \pi ^+$	$4.106 \pm 0.056$
${B_c^+} \rightarrow {J/\psi } {D_s^+}$	$1.849 \pm 0.034$
${B_c^+} \rightarrow {J/\psi } {D_s^{*+}}$, $A_{00}$	$1.829 \pm 0.053$
${B_c^+} \rightarrow {J/\psi } {D_s^{*+}}$, $A_{\pm \pm }$	$1.712 \pm 0.035$

The ratio ${\mathcal {R}}_{{D_s^{*+}}/{D_s^+}}$ is calculated as

4 $$\begin{aligned} {\mathcal {R}}_{{D_s^{*+}}/{D_s^+}}= & {} \frac{{\mathcal {B}}_{{B_c^+} \rightarrow {J/\psi } {D_s^{*+}}}}{{\mathcal {B}}_{{B_c^+} \rightarrow {J/\psi } {D_s^+}}}\nonumber \\= & {} \frac{N_{{B_c^+} \rightarrow {J/\psi } {D_s^{*+}}}}{N_{{B_c^+} \rightarrow {J/\psi } {D_s^+}}} \times \frac{{\mathcal {A}}_{{B_c^+} \rightarrow {J/\psi } {D_s^+}}}{{\mathcal {A}}_{{B_c^+} \rightarrow {J/\psi } {D_s^{*+}}}}, \end{aligned}$$

where the ratio of the yields ${N_{{B_c^+} \rightarrow {J/\psi } {D_s^{*+}}}}/{N_{{B_c^+} \rightarrow {J/\psi } {D_s^+}}}$ and its uncertainty is extracted from the fit as a parameter in order to account for correlations between the yields.

The fraction of the $A_{\pm \pm }$ component contribution in ${B_c^+} \rightarrow {J/\psi } {D_s^{*+}}$ decay is calculated from the ${f_{\pm \pm }}$ value quoted in Table 1 by applying a correction to account for the different acceptances for the two component contributions:

5 $$\begin{aligned} \Gamma _{\pm \pm }/\Gamma = {f_{\pm \pm }}\times \frac{{\mathcal {A}}_{{B_c^+} \rightarrow {J/\psi } {D_s^{*+}}}}{{\mathcal {A}}_{{B_c^+} \rightarrow {J/\psi } {D_s^{*+}},{\mathcal {A}}_{\pm \pm }}}. \end{aligned}$$

## Systematic uncertainties

The systematic uncertainties of the measured values are determined by varying the analysis procedure and repeating all calculations. Although some sources can have rather large effects on the individual decay rate measurements, they largely cancel in the ratios of the branching fractions due to correlation between the effects on the different decay modes. The following groups of systematic uncertainties are considered.

The first group of sources of systematic uncertainty relates to possible differences between the data and simulation affecting the acceptances for the decay modes. Thus, an effect of the $${B_c^+} $$ production model is evaluated by varying the simulated $${p_{\text {T}}} $$ and $$|\eta |$$ spectra while preserving agreement with the data distributions obtained using the abundant $${B_c^+} \rightarrow {J/\psi } \pi ^+$$ channel. These variations have very similar effects on the acceptances for the different decay modes, thus giving rather moderate estimates of the uncertainties, not exceeding 3 % in total, on the ratios of branching fractions. The effect of presence of other $${D_s^+} $$ decay modes with $$(K^+K^-\pi ^+)$$ final state on the calculated acceptances is studied with a separate MC simulation. Its conservative estimate yields 0.4 % which is assigned as $${\mathcal {R}}_{{D_s^+}/\pi ^+}$$ and $${\mathcal {R}}_{{D_s^{*+}}/\pi ^+}$$ uncertainties. An uncertainty on the tracking efficiency is dominated by the uncertainty of the detector material description in the MC simulation. Samples generated with distorted geometries and with increased material are used to estimate the effect on track reconstruction efficiencies. When propagated to the ratios of branching fractions, these estimates give 0.5 % uncertainty for $${\mathcal {R}}_{{D_s^+}/\pi ^+}$$ and $${\mathcal {R}}_{{D_s^{*+}}/\pi ^+}$$ due to the two extra tracks in $${B_c^+} \rightarrow {J/\psi } {D_s^{(*)+}} $$ modes. Limited knowledge of the $${B_c^+} $$ and $${D_s^+} $$ lifetimes leads to an additional systematic uncertainty. The simulated proper decay times are varied within one standard deviation from the world average values [22] resulting in uncertainties of $$\sim $$1 % assigned to $${\mathcal {R}}_{{D_s^+}/\pi ^+}$$ and $${\mathcal {R}}_{{D_s^{*+}}/\pi ^+}$$ due to the $${B_c^+} $$ lifetime, and 0.3 % due to the $${D_s^+} $$ lifetime. Removing the requirement on $${p_{\text {T}}} ({B_c^+})/\sum {p_{\text {T}}} (\mathrm {trk})$$ is found to produce no noticeable effect on the measured values.

The next group of uncertainties originates from the signal extraction procedure. These uncertainties are evaluated separately for ${J/\psi } {D_s^+}$ and ${J/\psi } \pi ^+$ candidate fits. For the former, the following variations of the fit model are applied and the difference is treated as a systematic uncertainty:

**Table 4:** Relative systematic uncertainties on the measured ratios of branching fractions $R_{{D_s^+}/\pi ^+}$, $R_{{D_s^{*+}}/\pi ^+}$, $R_{{D_s^{*+}}/{D_s^+}}$ and on the transverse polarisation fraction $\Gamma _{\pm \pm }/\Gamma$

Source	Uncertainty (%)			
	$R_{{D_s^+}/\pi ^+}$	$R_{{D_s^{*+}}/\pi ^+}$	$R_{{D_s^{*+}}/{D_s^+}}$	$\Gamma _{\pm \pm }/\Gamma$
Simulated ${p_{\text {T}}} ({B_c^+})$ spectrum	0.4	0.9	0.5	0.4
Simulated $|\eta ({B_c^+})|$ spectrum	1.9	2.4	0.6	0.2
Other $${D_s^+} $$ decay modes contribution	0.4	0.4	–	–
Tracking efficiency	0.5	0.5	$<$0.1	$<$0.1
$${B_c^+} $$ lifetime	1.2	1.3	$$<$$0.1	$$<$$0.1
$${D_s^+} $$ lifetime	0.3	0.3	$$<$$0.1	$$<$$0.1
${B_c^+} \rightarrow {J/\psi } {D_s^{(*)+}}$ signal extraction	4.4	10.5	10.7	17.4
${B_c^+} \rightarrow {J/\psi } \pi ^+$ signal extraction	8.5	8.5	–	–
$${D_s^{*+}} $$ branching fractions	$$<$$0.1	$$<$$0.1	$$<$$0.1	1.1
MC sample sizes	2.3	2.4	2.7	2.2
Total	10.1	14.0	11.0	17.6
${\mathcal {B}}_{{D_s^+} \rightarrow \phi (K^+K^-)\pi ^+}$	5.9	5.9	–	–

- different background mass shape parametrisations (three-parameter exponential, second- and third-order polynomials), different fitted mass range (reduced by up to 40 MeV from each side independently);
- a double Gaussian or double-sided Crystal Ball function [27–29] for ${B_c^+} \rightarrow {J/\psi } {D_s^+}$ signal description; variation of the modified Gaussian width within 10 % of the MC simulation value;
- variation of the smoothness of the ${B_c^+} \rightarrow {J/\psi } {D_s^{*+}}$ signal mass templates, which is controlled by a parameter of the kernel estimation procedure [25];
- similar variation of the smoothness of the ${B_c^+} \rightarrow {J/\psi } {D_s^{(*)+}}$ signal angular templates;
- variation of the smoothness of the sideband templates used for the background angular PDF construction; different ranges of the sidebands; different sideband interpolation procedure;
- modelling of the correlation between the mass and angular parts of the signal PDFs. This correlation takes place only at the detector level and manifests itself in degradation of the mass resolution for higher values of $|\cos \theta ^\prime (\mu ^+)|$. A dedicated fit model accounting for this effect is used for the data fit. The impact on the result is found to be negligible compared to the total uncertainty.

The first two items give the dominant contributions to the uncertainties of the ratios of branching fractions while the transverse polarisation fraction measurement is mostly affected by the background angular modelling variations. For the normalisation channel fit model, the similar variations of the background and signal mass shape parametrisation are applied. The deviations produced by the variations of the fits reach values as high as 10–15 % thus making them the dominant sources of systematic uncertainty.

The branching fractions of $${D_s^{*+}} $$ [22] are varied in simulation within their uncertainties to estimate their effect on the measured quantities. Very small uncertainties are obtained for the $${\mathcal {R}}_{{D_s^{*+}}/\pi ^+}$$ and $${\mathcal {R}}_{{D_s^{*+}}/{D_s^+}}$$, while for $$\Gamma _{\pm \pm }/\Gamma $$, the estimate is $$\sim $$1 %.

The statistical uncertainties on the acceptance values due to the MC sample sizes are also treated as a separate source of systematic uncertainty and estimated to be 2–3 %.

In order to check for a possible bias from using three-muon triggers, vetoing the $${D_s^+} $$ meson daughter tracks identified as muons is tested and found not to affect the measurement.

Finally, since ${\mathcal {B}}_{{D_s^+} \rightarrow \phi (K^+K^-)\pi ^+}$ enters Eq. (2), its uncertainty, evaluated from Ref. [26] as 5.9 %, is propagated to the final values of the relative branching fractions.

The systematic uncertainties on the measured quantities are summarised in Table 4.

## Results

The following ratios of the branching fractions are measured:

6 $$\begin{aligned} {\mathcal {R}}_{{D_s^+}/\pi ^+}&= \frac{{\mathcal {B}}_{{B_c^+} \rightarrow {J/\psi } {D_s^+}}}{{\mathcal {B}}_{{B_c^+} \rightarrow {J/\psi } \pi ^+}}\nonumber \\&= 3.8 \pm 1.1\text{(stat.) } \pm 0.4\text{(syst.) } \pm 0.2\text{(BF) },\\ {\mathcal {R}}_{{D_s^{*+}}/\pi ^+}&= \frac{{\mathcal {B}}_{{B_c^+} \rightarrow {J/\psi } {D_s^{*+}}}}{{\mathcal {B}}_{{B_c^+} \rightarrow {J/\psi } \pi ^+}} \nonumber \\&= 10.4 \pm 3.1\text{(stat.) } \pm 1.5\text{(syst.) } \pm 0.6\text{(BF) },\\ {\mathcal {R}}_{{D_s^{*+}}/{D_s^+}}&= \frac{{\mathcal {B}}_{{B_c^+} \rightarrow {J/\psi } {D_s^{*+}}}}{{\mathcal {B}}_{{B_c^+} \rightarrow {J/\psi } {D_s^+}}}\nonumber \\&= 2.8^{+1.2}_{-0.8}\text{(stat.) } \pm 0.3\text{(syst.) }, \end{aligned}$$

where the BF uncertainty corresponds to the knowledge of ${\mathcal {B}}_{{D_s^+} \rightarrow \phi (K^+K^-)\pi ^+}$. The relative contribution of the $A_{\pm \pm }$ component in ${B_c^+} \rightarrow {J/\psi } {D_s^{*+}}$ decay is measured to be

7 $$\begin{aligned} \Gamma _{\pm \pm }/\Gamma = 0.38 \pm 0.23\text{(stat.) } \pm 0.07\text{(syst.) } \end{aligned}$$

**Fig. 7:**
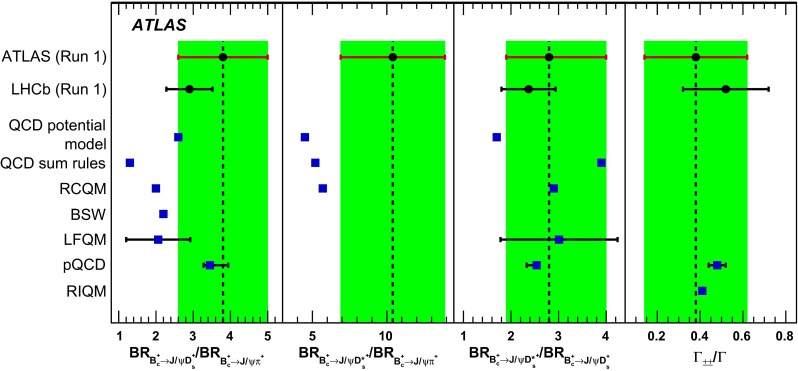
Comparison of the results of this measurement with those of LHCb [10] and theoretical predictions based on a QCD relativistic potential model [3], QCD sum rules [4], relativistic constituent quark model (RCQM) [5], BSW relativistic quark model (with fixed average transverse quark momentum $\omega =0.40$ GeV) [6], light-front quark model (LFQM) [7], perturbative QCD (pQCD) [8], and relativistic independent quark model (RIQM) [9]. The uncertainties of the theoretical predictions are shown if they are explicitly quoted in the corresponding papers. Statistical and systematic uncertainties added in quadrature are quoted for the results of ATLAS and LHCb.

These results are compared with those of the LHCb measurement [10] and to the expectations from various theoretical calculations in Table 5 and Fig. 7. The measurement agrees with the LHCb result. All ratios are well described by the recent perturbative QCD predictions [8]. The expectations from models in Refs. [3, 5, 7] as well as the sum-rules prediction [4] for the ratio ${\mathcal {R}}_{{D_s^{*+}}/{D_s^+}}$ are consistent with the measurement. The QCD relativistic potential model predictions [3] are consistent with the measured ${\mathcal {R}}_{{D_s^+}/\pi ^+}$ ratio while the expectations from the sum rules [4] and models in Refs. [5–7] are somewhat smaller than the measured value. The predictions in Refs. [3–5, 7] are also generally smaller than the measured ratio ${\mathcal {R}}_{{D_s^{*+}}/\pi ^+}$; however, the discrepancies do not exceed two standard deviations when taking into account only the experimental uncertainty.

**Table 5:** Comparison of the results of this measurement with those of LHCb [10] and theoretical predictions based on a QCD relativistic potential model [3], QCD sum rules [4], relativistic constituent quark model (RCQM) [5], BSW relativistic quark model (with fixed average transverse quark momentum $\omega =0.40$ GeV) [6], light-front quark model (LFQM) [7], perturbative QCD (pQCD) [8], and relativistic independent quark model (RIQM) [9]. The uncertainties of the theoretical predictions are shown if they are explicitly quoted in the corresponding papers. Statistical and systematic uncertainties added in quadrature are shown for the results of ATLAS and LHCb

${\mathcal {R}}_{{D_s^+}/\pi ^+}$	${\mathcal {R}}_{{D_s^{*+}}/\pi ^+}$	${\mathcal {R}}_{{D_s^{*+}}/{D_s^+}}$	$\Gamma _{\pm \pm }/\Gamma$	Ref.
$3.8 \pm 1.2$	$10.4 \pm 3.5$	$2.8^{+1.2}_{-0.9}$	$0.38 \pm 0.24$	ATLAS
$2.90\pm 0.62$	–	$2.37\pm 0.57$	$0.52 \pm 0.20$	LHCb [10]
2.6	4.5	1.7	–	QCD potential model [3]
1.3	5.2	3.9	–	QCD sum rules [4]
2.0	5.7	2.9	–	RCQM [5]
2.2	–	–	–	BSW [6]
$2.06\pm 0.86$	–	$3.01\pm 1.23$	–	LFQM [7]
$3.45^{+0.49}_{-0.17}$	–	$2.54^{+0.07}_{-0.21}$	$0.48\pm 0.04$	pQCD [8]
–	–	–	0.410	RIQM [9]

The measured fraction of the $A_{\pm \pm }$ component agrees well with the prediction of the relativistic independent quark model [9] and perturbative QCD [8].

## Conclusion

A study of ${B_c^+} \rightarrow {J/\psi } {D_s^+}$ and ${B_c^+} \rightarrow {J/\psi } {D_s^{*+}}$ decays has been performed. The ratios of the branching fractions ${\mathcal {B}}_{{B_c^+} \rightarrow {J/\psi } {D_s^+}}/{\mathcal {B}}_{{B_c^+} \rightarrow {J/\psi } \pi ^+}$, ${\mathcal {B}}_{{B_c^+} \rightarrow {J/\psi } {D_s^{*+}}}/{\mathcal {B}}_{{B_c^+} \rightarrow {J/\psi } \pi ^+}$, ${\mathcal {B}}_{{B_c^+} \rightarrow {J/\psi } {D_s^{*+}}}/{\mathcal {B}}_{{B_c^+} \rightarrow {J/\psi } {D_s^+}}$ and the transverse polarisation fraction of ${B_c^+} \rightarrow {J/\psi } {D_s^{*+}}$ decay have been measured by the ATLAS experiment at the LHC using *pp* collision data corresponding to an integrated luminosity of 4.9 fb$^{-1}$ at 7 TeV centre-of-mass energy and 20.6 fb$^{-1}$ at 8 TeV. The polarisation is found to be well described by the available theoretical approaches. The measured ratios of the branching fraction are generally described by perturbative QCD, sum rules, and relativistic quark models. There is an indication of underestimation of the decay rates for the ${B_c^+} \rightarrow {J/\psi } {D_s^{(*)+}}$ decays by some models, although the discrepancies do not exceed two standard deviations when taking into account only the experimental uncertainty. The measurement results agree with those published by the LHCb experiment.

## Footnotes

1 Charge conjugate states are implied throughout the paper unless otherwise stated.

2 ATLAS uses a right-handed coordinate system with its origin at the nominal interaction point (IP) in the centre of the detector and the *z*-axis along the beam pipe. The *x*-axis points from the IP to the centre of the LHC ring, and the *y*-axis points upward. Cylindrical coordinates $(r,\phi )$ are used in the transverse plane, $\phi$ being the azimuthal angle around the beam pipe. The pseudorapidity is defined in terms of the polar angle $\theta$ as $\eta =-\ln \tan (\theta /2)$.

3 Using a system of units with $c = 1$ is implied throughout the paper.

4 The transverse decay length of a particle is defined as the transverse distance between the production (primary) vertex and the particle decay (secondary) vertex projected along its transverse momentum.
